# Renal Medullary and Cortical Correlates in Fibrosis, Epithelial Mass, Microvascularity, and Microanatomy Using Whole Slide Image Analysis Morphometry

**DOI:** 10.1371/journal.pone.0161019

**Published:** 2016-08-30

**Authors:** Alton B. Farris, Carla L. Ellis, Thomas E. Rogers, Diane Lawson, Cynthia Cohen, Seymour Rosen

**Affiliations:** 1 Emory University, Department of Pathology, Atlanta, Georgia, United States of America; 2 Harvard University/ Beth Israel Deaconess Medical Center, Department of Pathology, Boston, Massachusetts, United States of America; Johns Hopkins School of Medicine, UNITED STATES

## Abstract

Renal tubulointerstitial injury often leads to interstitial fibrosis and tubular atrophy (IF/TA). IF/TA is typically assessed in the renal cortex and can be objectively quantitated with computerized image analysis (IA). However, the human medulla accounts for a substantial proportion of the nephron; therefore, medullary scarring will have important cortical consequences and may parallel overall chronic renal injury. Trichrome, periodic acid–Schiff (PAS), and collagen III immunohistochemistry (IHC) were visually examined and quantitated on scanned whole slide images (WSIs) (N = 67 cases). When tuned to measure fibrosis, IA of trichrome and Trichrome-PAS (T-P) WSIs correlated for all anatomic compartments (among cortex, medulla, and entire tissue, r = 0.84 to 0.89, P all <0.0001); and collagen III deposition correlated between compartments (r = 0.69 to 0.89, P <0.0001 to 0.0002); however, trichrome and T-P measures did not correlate with collagen deposition, suggesting heterogeneous contributions to extracellular matrix deposition. Epithelial cell mass (EPCM) correlated between cortex and medulla when measured with cytokeratin IHC and with the trichrome red portion (r = 0.85 and 0.66, respectively, all P < 0.0001). Visual assessment also correlated between compartments for fibrosis and EPCM. Correlations were found between increasing medullary inner stripe (IS) width and fibrosis in all of the tissue and the medulla by trichrome morphometry (r = 0.56, P < 0.0001, and r = 0.48, P = 0.00008, respectively). Weak correlations were found between increasing IS width and decreasing visual assessment of all tissue EPCM. Microvessel density (MVD) and microvessel area (MVA) measured using a MVD algorithm applied to CD34 IHC correlated significantly between all compartments (r = 0.76 to 0.87 for MVD and 0.71 to 0.87 for MVA, P all < 0.0001). Overall, these findings demonstrate the interrelatedness of the cortex and medulla and the importance of considering the renal parenchyma as a whole.

## Introduction

The assessment of renal tubulointerstitial injury is important for both native and transplant kidneys; however, past studies, including some of our own studies,[1, 2] have primarily focused on the renal cortex; and traditionally, renal tubulointerstitial injury assessment is often restricted to the cortex. This, of course, ignores an area critical to proper renal function and, furthermore, does not provide the basis for understanding of the "medullary only " biopsy.[3] Indeed, the usual renal biopsy does consist primarily of cortex, but commonly a portion of outer medulla is included. The outer medulla has two major zones: outer stripe (OS) and inner stripe (IS). The OS cellular mass is mostly formed by proximal tubules and is very thin or even sometimes considered negligible in the normal human kidney in contrast to the rat/mouse. The IS consists of an epithelial cell mass (EPCM) mostly formed by thick ascending limbs and collecting ducts. Recognition of these zones in the diseased renal biopsy depends on multiple factors including defining the corticomedullary junction connective tissue/vascular components, the appropriate epithelial content, and the presence of vasa recta. With increasing injury, EPCM diminishes in concert with thick ascending limb tubular atrophy/loss (TA), but with preservation of collecting ducts.[4–12]

Chronic renal diseases result in interstitial extracellular matrix accumulation, referred to as “fibrosis”, which can eventually contribute to renal failure.[13–19] Visual assessment using trichrome stained slides is often used in fibrosis morphometry.[20–22] Immunohistochemistry (IHC) for type III collagen can also be used.[23–25] Studies have demonstrated that collagen III staining methods can be predictive of decreased glomerular filtration rate (GFR).[23–25]

To study medullary fibrotic injury, interstitial fibrosis (IF), tubular basement membrane (BM) area, epithelial mass, and microvessel density were quantitated in both the renal cortex and medulla in a morphometric study visually and by image analysis. Using these techniques, we focused on the cortex, medulla, OS, and IS to better characterize injury in these regions.

## Materials and Methods

### Biopsy samples

For consecutive native renal biopsies (N = 64), the sole inclusion criterion was that medullary tissue be available for evaluation (primarily outer medulla). Sections from 3 nephrectomy specimens were also included that had minimal fibrosis on morphologic inspection and were obtained from non-neoplastic parenchyma from nephrectomies performed for carcinoma. Additional data regarding the specimens are included in S6 Table. The studies described here have been approved by our Institutional Review Board (IRB) (Emory University IRB00055904), which determined that written, informed consent was not needed since the retrospective study was conducted on excess archival material and had no influence on patient care.

### Stains

Trichrome and periodic acid Schiff (PAS) using standard histologic techniques. Tissue was also stained with antibodies to collagen III (BioGenex, San Ramon, CA; Monoclonal clone HWD1.1; at 1:80 titration), cytokeratin AE1/AE3 (Dako, Carpinteria, CA; prediluted), Cytokeratin CAM 5.2 (BD Biosciences, San Jose, CA; prediluted) cytokeratin cocktail (AE1/AE3;CAM5.2), cytokeratin MNF-116 (Dako; at 1/100), and CD34 (Dako; Monoclonal clone QBEND; at 1:320 titration). IHC was performed with automated stainers similar to methods previously published [1, 26].

### Morphometry

Stained sections were scanned with an Aperio ScanScope XT (Aperio Technologies, Inc., Vista, CA) to obtain whole slide images (WSIs). The Aperio Scanscope allowed scanning and quantitation of the whole slide using a 20X objective lens with a numerical aperture of 0.75 coupled with a doubler objective to achieve a scan of whole slides at 40X magnification.

**Fig 1:**
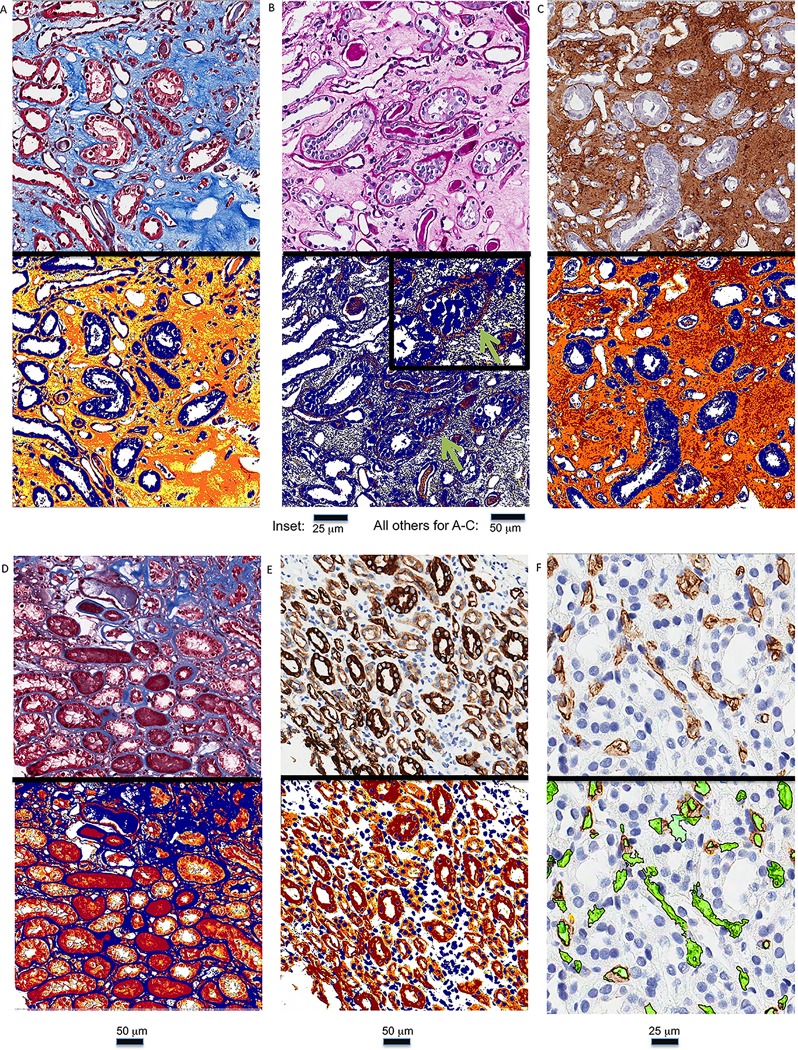
Whole slide image (WSI) quantitation is shown for (A) fibrosis using trichrome, (B) basement membrane mass using Periodic acid–Schiff (PAS), (C) fibrosis using collagen III immunohistochemistry, (D) epithelial mass using the red portion of the trichrome, (E) epithelial mass using cytokeratin immunohistochemistry, and (F) using CD34 immunohistochemistry. In (A)-(F), the WSI without quantitation is seen in the upper panel; and a WSI quantitation markup image is shown in the lower panels in which yellow/orange/red areas are counted using a positive pixel count algorithm for (A)-(E) and the microvessel density is quantitated by a microvessel density algorithm in green in (F). The inset in (B) depicts the quantitation of a tubular basement membrane (green arrow) at higher power.

For trichrome, PAS, cytokeratin, and collagen III measurements, stained sections were scanned with an Aperio ScanScope XT and analyzed using the ImageScope Positive Pixel Count (PPC) algorithm (Fig 1).[27, 28] The PPC algorithm default parameters adequately detected the brown chromogen of the collagen III and cytokeratin immunohistochemistry (hue of 0.1 and width of 0.5). For trichrome and PAS, hue values for blue and pink were measured in all cases, and an average hue for the trichrome (0.60) and PAS (0.86) were used when evaluating these stains. The default hue width was used for the trichrome stained slides, and a narrower hue width (0.05) was selected for the PAS stained slides to set an adequate threshold for only detecting PAS positive basement membranes, yielding the % of tissue composed of BM material. In this manner, the % cortical interstitial fibrosis (Trichrome%–PAS%, herein also referred to as “T-P fibrosis”) was calculated (% positive trichrome pixels minus % positive PAS pixels). For the entire cortex and entire medulla, EPCM was quantitated using a PPC algorithm tuned to detect cytokeratin brown and trichrome red (Fig 1). To detect EPCM using the red of the trichrome stain, the reddish hue of the Biebrich scarlet was selected (0.96) and delineated epithelium relatively effectively when the hue width of 0.20 was used in the PPC algorithm. In cases where more than 1 cytokeratin was used, an average of the cytokeratin quantitation was used.

Microvascularity was quantitated on CD34-stained slides with the Aperio microvessel density (MVD) algorithm, which provided a measure of MVD and mean vascular area (MVA) (Fig 1). Our group has previously used this algorithm in the assessment of microvascularity, and the same methods were used in this study [26, 29]. The algorithm also provided measures of vascular wall thickness and vascular perimeter [in data not included in this study]. We manually excluded glomeruli when conducting MVD and MVA analysis.

### Visual Assessment

Pathologist visual assessment was performed by viewing WSIs on a computer monitor, and the following parameters were recorded by the first author (A.B.F.): % of tissue composed of fibrous tissue on trichrome-stained slides (visual % interstitial fibrosis), % EPCM based on the red-stained portion of the trichrome-stained slides, and % of tubules atrophic on PAS-stained slides (% TA). The proportion of tissue composed by cortex and medulla were also visually estimated, revealing that the cases were composed of 45.4 ± 26.0% cortex and 54.6 ± 26.0% medulla (mean ± standard deviation [SD]). The cortex was assessed in that part bounded by glomeruli, which eliminated the immediate subcapsular area and the larger vessels at the corticomedullary junction. The medulla was taken as the remainder of the tissue, and the medulla was further separated into the outer stripe (OS) and the inner (IS) stripe for purposes of measuring the widths of the OS and IS based on the differing appearances of the tubules in the OS and IS regions. The OS tubules tended to have more copious cytoplasm and were more distinct than the IS tubules; and based on this feature, the OS and IS width were measured using a digital ruler in the ImageScope program.

### Renal functional data

Creatinine values and patient parameters were obtained from clinical databases.

### Statistical Analysis

Microsoft Excel (Microsoft Corporation, Redmond, WA) was used to record data and perform preliminary statistical analysis. Further statistical analysis, including multivariate regression analysis and hierarchical clustering analysis, was performed in SAS JMP version 12.0.0 (SAS Institute, Cary, NC). We interpreted the data according guidelines that have been suggested for the interpretation of correlation r values in which correlation is rated as follows: r = 0.90 to 1.0, “very high”; r = 0.70 to 0.90, “high”, r = 0.50 to 0.70, “moderate”; r = 0.30 to 0.50, “low”; and r = 0.00 to 0.30, “negligible” correlation.[30, 31]

## Results

### Anatomic Features Assessed

The stains employed in these studies could be used to highlight different anatomic features and pathologic processes, as shown in Fig 1. Fibrotic areas could be detected with the blue of the trichrome stain and with collagen III IHC. The PAS stain and detection algorithm was able to detect tubular basement membranes, and as alluded to previously, the result from the PAS detection algorithm was subtracted from the trichrome algorithm to give a measure of fibrosis (i.e., Trichrome–PAS, hereafter referred to as “T-P”). Trichrome and PAS stains both stained proteinaceous casts in a number of cases; therefore, the T-P method was sometimes effective in subtracting the proteinaceous casts. EPCM could be detected with the red of the trichrome stain and with cytokeratin IHC. Microvascularity was highlighted with CD34 IHC. Since these anatomic features and pathologic processes could be appreciated in a qualitative sense in this manner, computerized algorithms could be used to quantitate the staining, as shown in Table 1 and discussed below.

**Table 1:** Summary of measurements obtained is shown for various measures of interstitial fibrosis, tubular atrophy, epithelial cell mass [EPCM, Epithel below], microvessel density (MVD), and microvessel area (MVA). Stains applied include trichrome (Tri), periodic acid–Schiff (PAS), collagen III (Col) immunohistochemistry (IHC), cytokeratin (CK) IHC for EPCM, and CD34 IHC for the MVD and MVA. Measurements are either performed using morphometric or visual (Vis) methods. For fibrosis morphometry, a method employing trichrome minus the PAS measurement is used (T-P). For EPCM, the “Red” of the trichrome is also used (RedTri). The outer (Out) and inner (Inn) stripe (Stri) widths are also measured. (Std Dev = Standard Deviation).

Measurement	N	Mean	Std Dev	Minimum	Maximum
All-Tri (%)	63	40.2	11.2	15.8	60.8
All-RedTri (%)	62	53.0	11.7	33.3	78.7
All-PAS (%)	61	16.4	4.7	0.7	34.6
All-T-P (%)	60	25.1	12.3	-11.0	58.4
Ctx-Tri (%)	62	31.7	10.3	10.3	52.9
Ctx-RedTri (%)	61	61.9	11.3	35.5	86.5
Ctx-PAS (%)	60	16.1	5.2	0.6	40.7
Ctx-T-P (%)	60	16.5	11.5	-22.8	36.1
Med-Tri (%)	63	47.0	12.0	15.6	72.0
Med-RedTri (%)	62	46.0	12.1	21.5	80.5
Med-PAS (%)	61	16.3	4.2	0.7	24.3
Med-T-P (%)	58	31.4	12.8	1.9	62.1
All-Col (%)	24	31.0	15.9	9.5	67.8
Ctx-Col (%)	24	20.4	12.8	6.9	52.8
Med-Col (%)	24	38.7	20.4	8.9	76.0
All-CKAvg (%)	58	52.9	18.2	8.9	83.5
Ctx-CKAvg (%)	56	58.6	12.6	30.4	85.0
Med-CKAvg (%)	58	55.6	12.0	31.3	81.4
Vis-All-Tri (%)	63	56.6	22.4	5	90
Vis-Ctx-Tri (%)	62	45.9	22.7	5	85
Vis-Med-Tri (%)	63	67.7	20.6	5	90
Vis-All-PAS-TA (%)	63	30.9	20.0	1	90
Vis-Ctx-PAS-TA (%)	62	31.8	21.1	1	90
Vis-Med-PAS-TA (%)	63	31.1	20.7	1	85
Vis-All-Tri-Epithel (%)	63	35.3	22.9	7	92
Vis-Ctx-Tri-Epithel (%)	62	47.9	25.0	10	95
Vis-Med-Tri-Epithel (%)	63	23.3	18.0	5	90
Out-Stri (mm)	62	1.8	0.8	0.1148	4.96
Inn-Stri (mm)	62	4.4	2.3	0.2508	14.857
All-MVD (vessels/um^2^)	64	0.000281	0.000206	0.0000285	0.000799
All-MVA (um^2^)	64	94.8	31.3	45.5632	168.475
Ctx-MVD (vessels/um^2^)	63	0.000367	0.000226	0.0000387	0.00113
Ctx-MVA (um^2^)	63	81.1	31.7	34.6286	200.93
Med-MVD (vessels/um^2^)	64	0.000346	0.000172	0.0000754	0.000769
Med-MVA (um^2^)	64	97.0	31.1	45.1734	189.77

**Fig 2:**
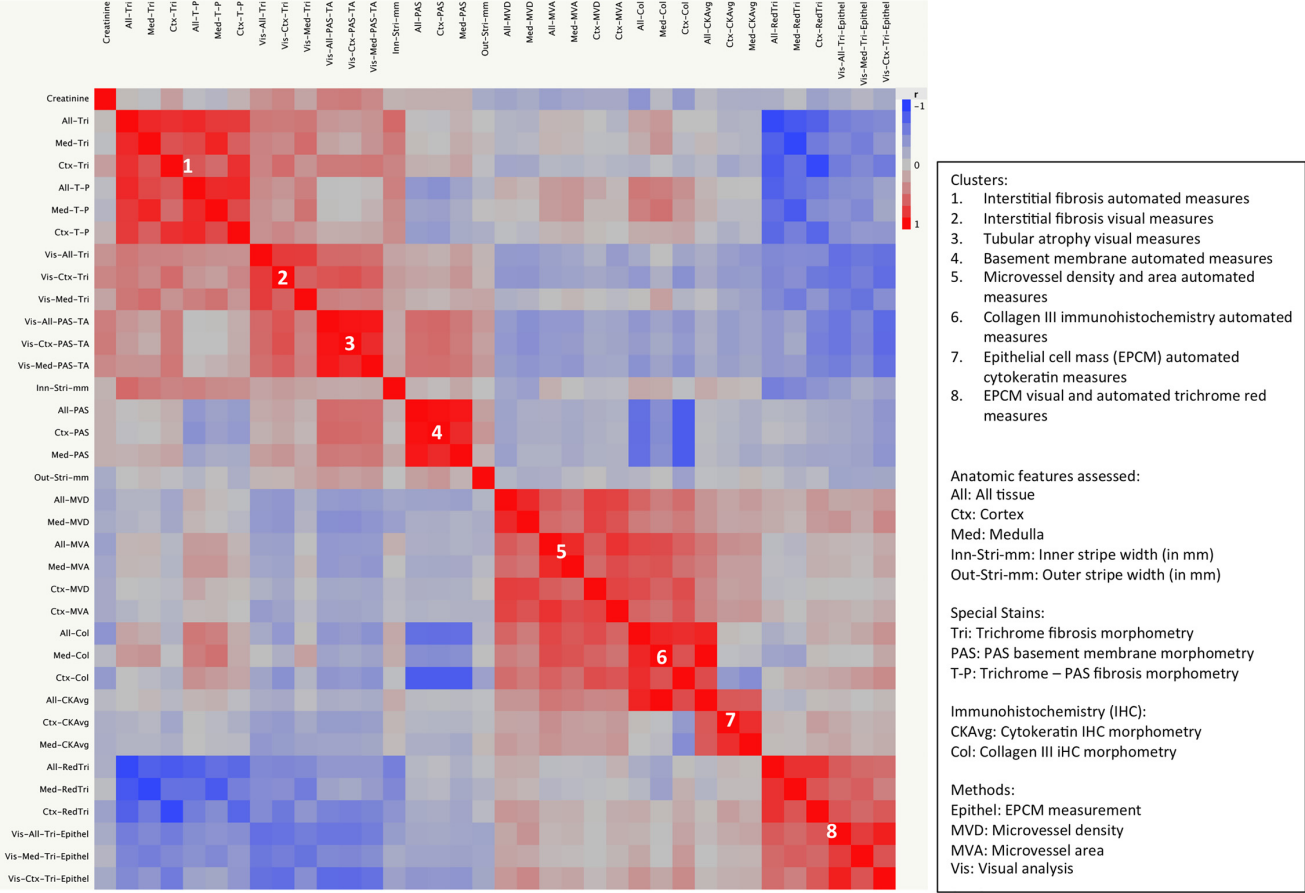
A color map of correlations between measured variables is shown. Variables that correlate closely are clustered.

Several relationships could be seen on a multivariate regression analysis of features measured. Groups that tended to correlate included the following in order of their interrelatedness: (1) interstitial fibrosis automated measures, (2) interstitial fibrosis visual measures, (3) tubular atrophy visual quantitation, (4) basement membrane automated quantitation, (5) microvessel density and area automated analysis, (6) collagen III IHC automated morphology, (7) EPCM automated cytokeratin measures, and (8) EPCM visual and automated trichrome red measures, based on a clustering of regressions that correlate shown in Fig 2. In addition, groups 1 and 2 are related; and this finding supports a correlation between the automated and visual interstitial fibrosis measures. Furthermore, it can be seen that groups 2 and 3 are correlated, demonstrating that interstitial fibrosis and tubular atrophy often occur in tandem. Lastly, groups 5 and 6 are correlated, showing that there is a relationship between the microvessel analysis measures and collagen III IHC. These correlations will be discussed in more detail below. Based on this analysis, it can also be seen that creatinine did not correlate tightly with any of the measured features. A two-way hierarchical clustering analysis depicting selected measures is shown in S1 Fig.

### Fibrosis Measurements

**Fig 3:**
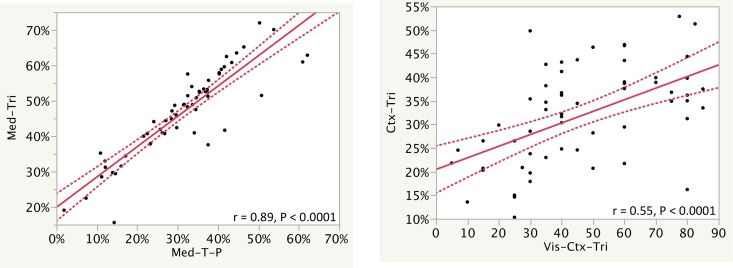
Correlation of selected fibrosis measures is shown. Image analysis of trichrome (Tri), trichrome analysis minus PAS analysis (T-P), and visual assessment (Vis) were assessed for all of the tissue, the cortex (Ctx), and the medulla (Med). Regression lines and r values of corresponding measurements show how measurements correlate. The curved dotted lines display the confidence limits for the expected mean value. Additional correlations are shown in S2 Fig.

Trichrome, periodic acid–Schiff (PAS), and Trichrome-PAS (T-P) interstitial fibrosis% showed a wide range of fibrosis positivity as measured by the PPC algorithm, from 16 to 61%, 0.7 to 35%, and -11 to 58% for all tissue, respectively. When tuned to measure fibrosis, image analysis of trichrome and T-P WSIs correlated for all anatomic compartments (r = 0.84–0.89, P all <0.0001). Visual measures of interstitial fibrosis also showed a wide range, from 5–90% for all tissue. For the same compartment, visual measures of interstitial fibrosis showed significant correlation with all of the trichrome and T-P measures (r = 0.37–0.55, P = 0.004 to < 0.0001). Furthermore, basement membrane staining, as measured by PAS morphometry, correlated between all anatomic compartments (r = 0.87–0.97, P all < 0.0001). A summary of the measures correlating between cortex and medulla are shown in Table 2. Key correlations are shown in Fig 3, and a more extended set of correlations is shown in S2 Fig and S1 Table.

**Table 2:** Measures are shown with regard to the strength of their correlation between cortex and medulla. Additional correlations are shown in S7 Table.

Measurement Method	Correlation r value^
**Highly correlated:**	
Cytokeratin IHC morphometry (CKAvg)	0.85
Periodic acid–Schiff (PAS) morphometry	0.87
PAS % tubular atrophy visual assessment (Vis-PAS-TA)	0.86
Microvessel density (MVD)	0.76
Trichrome–PAS (T-P) fibrosis morphometry	0.72
Microvessel area (MVA)	0.71
**Moderately correlated:**	
Collagen III IHC morphometry (Col)	0.69
Trichrome fibrosis morphometry (Tri)	0.68
Trichrome red epithelial staining visual assessment (Vis-Tri-Epithel)	0.67
Trichrome red epithelial staining morphometry (RedTri)	0.66
Trichrome fibrosis visual assessment (Vis-Tri)	0.60

^ The linear regression correlation r value is given; For all r values, P < 0.0001.

Col: collagen III immunohistochemistry (IHC), CKAvg: cytokeratin IHC for epithelial cell mass (EPCM), Epithel: EPCM assessment, MVD: microvessel density, MVA: microvessel area, PAS: periodic acid–Schiff, RedTri: EPCM using the “Red” of the trichrome, Tri: trichrome, T-P: fibrosis morphometry employing trichrome minus the PAS, and Vis: visual assessment.

### Tubular Atrophy

Visual assessment of tubular atrophy also correlated between compartments (r = 0.86 to 0.96, P all < 0.0001) as shown in S3 Fig and S2 Table. Visual assessment of tubular atrophy weakly correlated with interstitial fibrosis as measured by the trichrome morphometric method (r = 0.27 to 0.45, P = 0.04 to 0.0002) and also as evaluated by visual assessment of interstitial fibrosis (r = 0.42 to 0.68, P = 0.0005 to < 0.0001) as shown in S4 Fig.

### Collagen III Deposition

**Fig 4:**
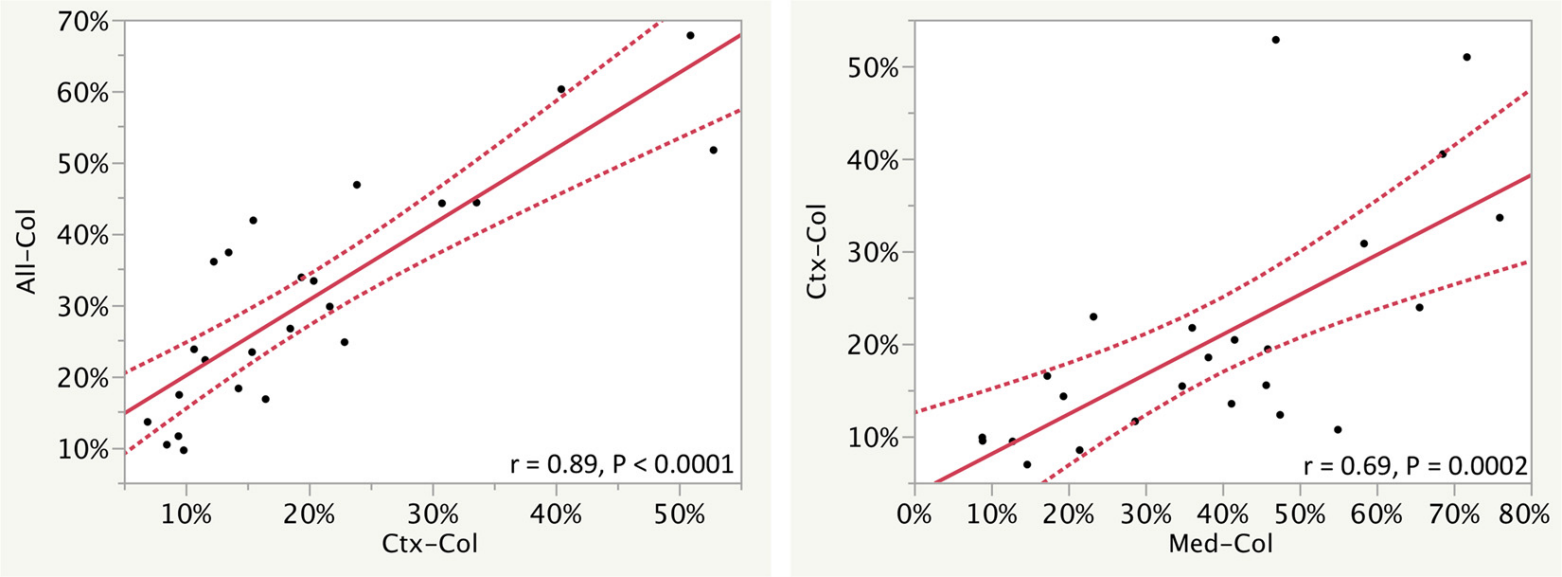
Selected correlation of fibrosis measures using collagen III immunohistochemistry (Col) are shown. Measurements were performed for all of the tissue, the cortex (Ctx), and the medulla (Med). Regression lines and r values of corresponding measurements show how measurements correlate. The curved dotted lines display the confidence limits for the expected mean value. Additional correlations are shown in S5 Fig.

Collagen III IHC showed a range of 9.5–68% for all tissue. Collagen III deposition correlated between compartments (r = 0.85, P < 0.0001 for all tissue vs. cortex, r = 0.89, P<0.0001 for all tissue vs. medulla, and r = 0.69, P = 0.0002 for cortex vs. medulla). Correlations are shown in Fig 4 and in extended format in S5 Fig. However, collagen III did not show a significant relationship with any of the other measures of interstitial fibrosis (i.e., trichrome, T-P, or visual measures).

### Epithelial Cell Mass

**Fig 5:**
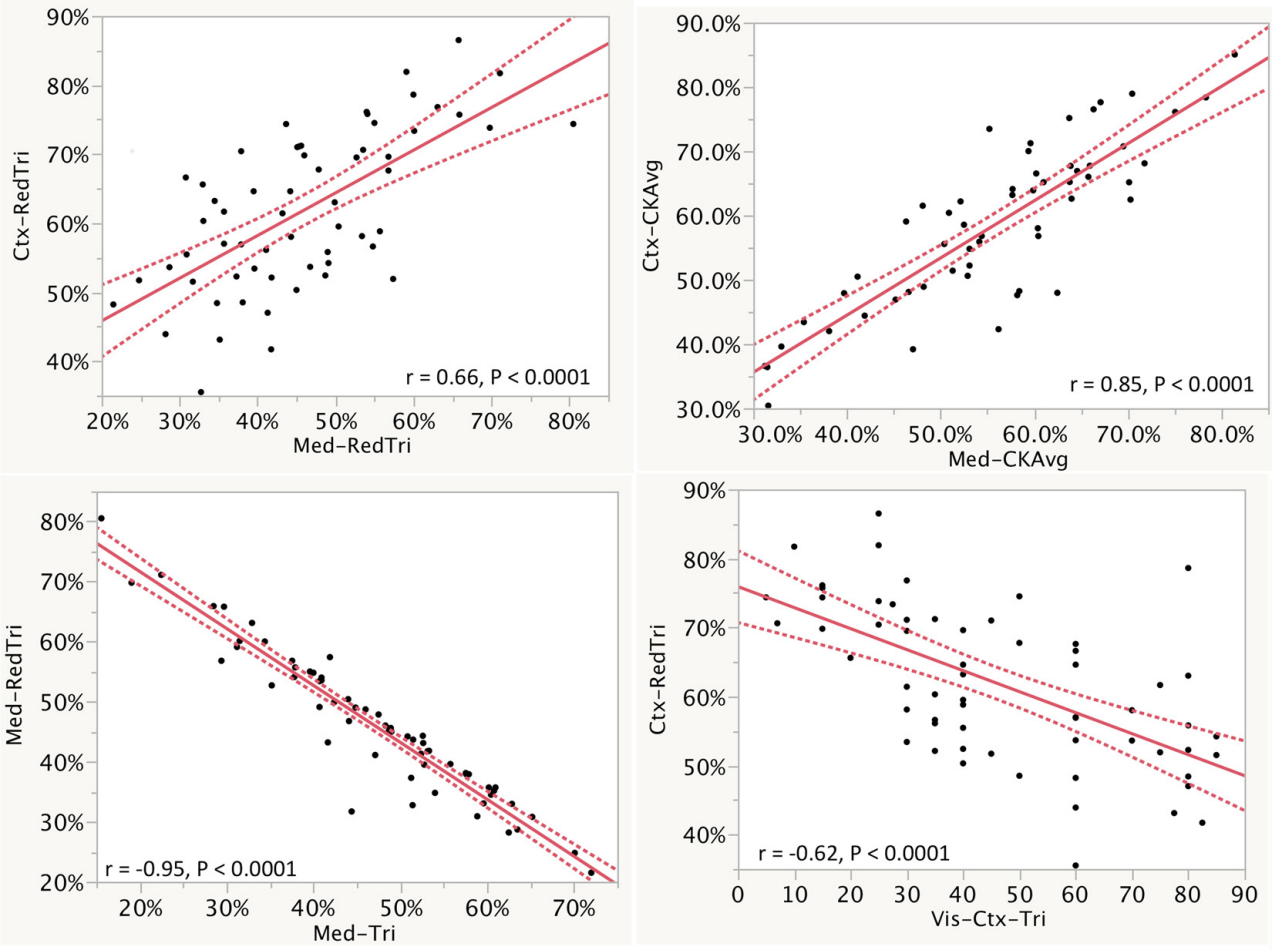
Selected correlation of epithelial cell mass measures are shown. Measurements were performed for all of the tissue, the cortex (Ctx), and the medulla (Med). Regression lines and r values of corresponding measurements show how measurements correlate. The curved dotted lines display the confidence limits for the expected mean value. Additional correlations are shown in S6 Fig.

The EPCM (mean ± standard deviation [SD]) of the cortex and medulla, respectively, were 62 ± 11% and 46 ± 12% on a measurement of the trichrome red staining portion; 59 ± 13% and 56 ± 12% on cytokeratin, and 48 ± 25% and 23 ± 18% on pathologist visual estimation of EPCM. Cortical and medullary EPCM declined together, with linear regression showing direct relationships between cortical and medullary EPCM by trichrome red staining (r = 0.66 between cortex and medulla, P < 0.0001) and cytokeratin immunohistochemistry (r = 0.85, P < 0.0001). For the same compartment (cortex, medulla, and all tissue), the measurement of EPCM using the trichrome red staining correlated with pathologist visual estimation of EPCM (r = 0.62 to 0.65, P all < 0.0001). For the same compartment, decreasing EPCM assessed using trichrome red staining correlated with measurement of increasing fibrosis using trichrome morphometry with a statistically significant inverse relationship (r = - 0.94 to—0.95, P all < 0.0001). A similar relationship was also seen with visual assessment of fibrosis on the trichrome with less tight but still statistically significant inverse correlations between decreasing EPCM and increasing fibrosis (r = -0.49 to -0.62, P all < 0.0001). Key correlations are shown in Fig 5 with additional correlations regarding EPCM in S6 Fig and S3 Table.

### Medullary Outer and Inner Stripe Width

**Fig 6:**
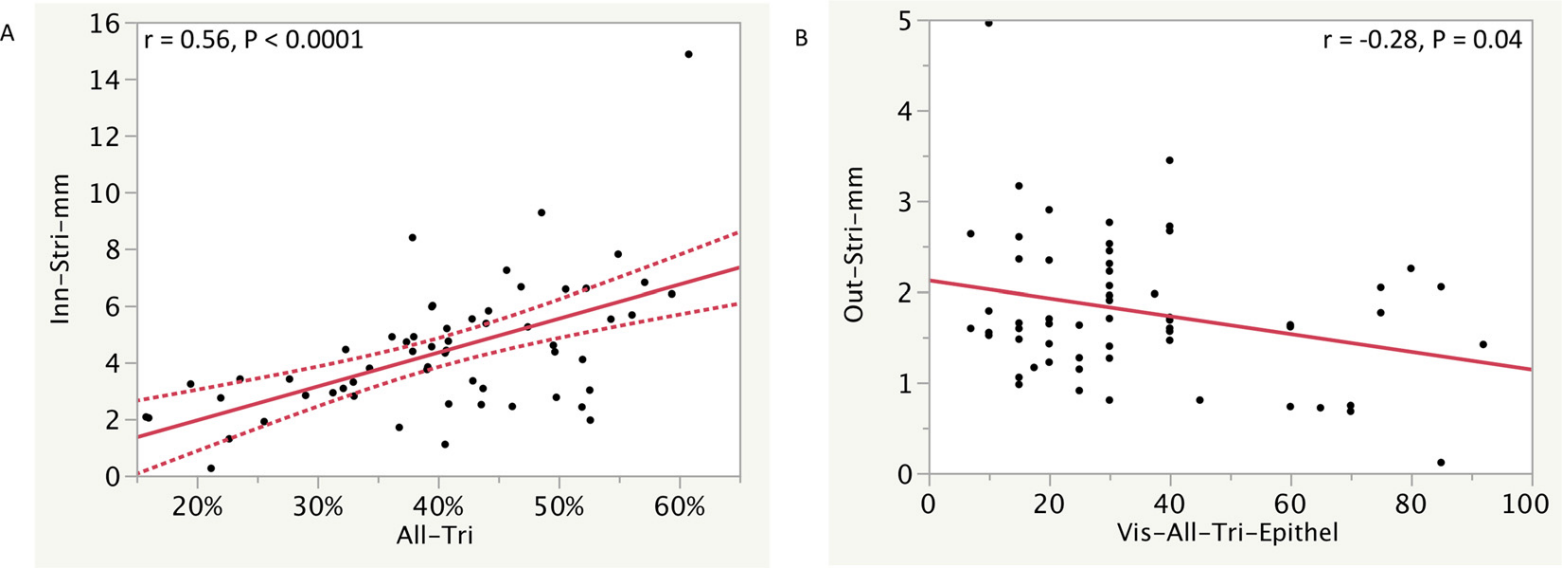
Correlations of microanatomic features are shown. (A) Correlation of outer and inner stripe width in millimeters (Out-Stri-mm and Inn-Stri-mm) with fibrosis measures, including all of the tissue, the cortex (Ctx), and the medulla (Med) using image analysis of trichrome (Tri). (B) Correlation of outer and inner stripe width in millimeters (Out-Stri-mm and Inn-Stri-mm) with measures of % epithelial cell mass [EPCM, Epithel below] by visual assessment. Regression lines and r values of corresponding measurements show how measurements correlate. The curved dotted lines display the confidence limits for the expected mean value. Additional correlations are shown in S7 Fig.

The medullary OS and IS width was 1.8 ± 0.8 and 4.4 ± 2.3 mm (mean ± SD), respectively. Statistically significant correlations were found between increasing IS width and fibrosis in all of the tissue by trichrome morphometry (r = 0.57, P < 0.0001) and medullary fibrosis by trichrome morphometry (r = 0.48, P = 0.00008). Weak but statistically significant correlations were found between increasing IS width and decreasing visual assessment of all tissue EPCM (r = -0.33, P = 0.01) and cortical EPCM (r = - 0.28, P = 0.02) and also between increasing OS width and decreasing visual assessment of all tissue EPCM (r = - 0.28, P = 0.04). Key correlations are shown in Fig 6 with additional correlations in S7 Fig and S4 Table.

### Microvascularity

**Fig 7:**
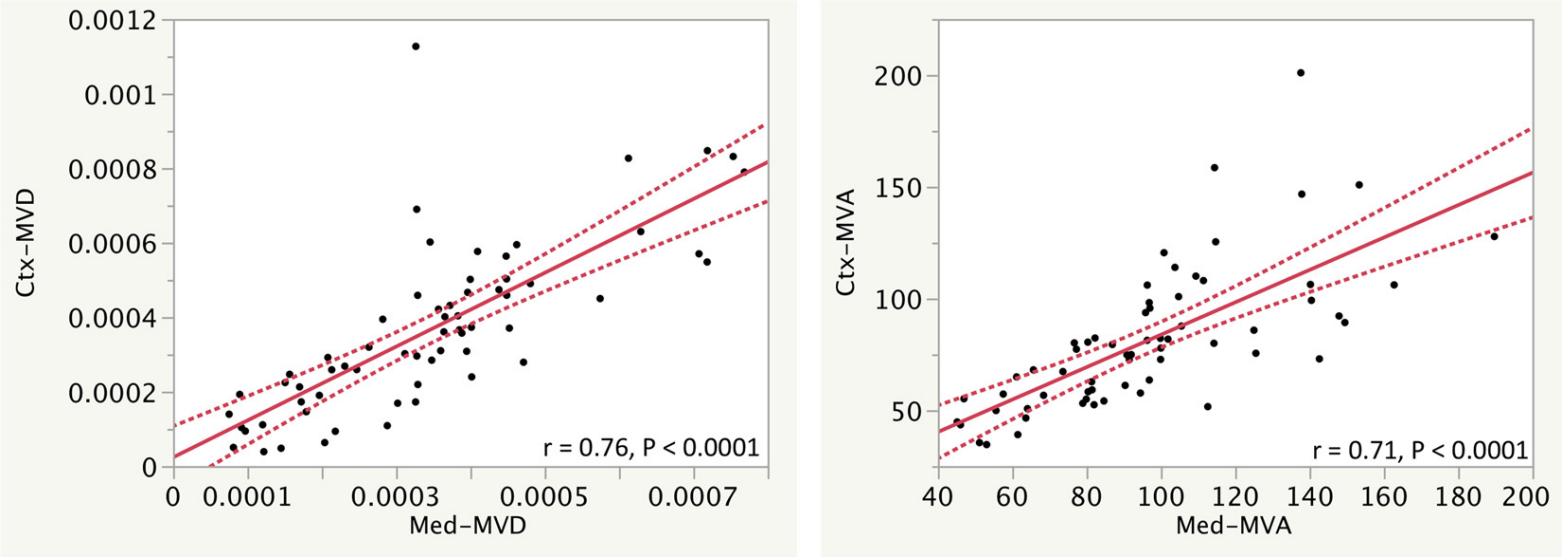
Correlation of measures of microvessel density (MVD in in vessels/um^2^) and mean vessel area (MVA in um^2^) are shown between the cortex (Ctx), and the medulla (Med). Regression lines and r values of corresponding measurements show how measurements correlate. The curved dotted lines display the confidence limits for the expected mean value. Additional correlations are shown in S8 Fig.

Tubulointerstitial microvascularity was highlighted with CD34 immunohistochemistry. The renal biopsies had heterogeneous disease, ranging from an essentially normal biopsy in which the MVD was 80x10^-5^ vessels/um^2^ in all of the tissue, diminishing to 2.8x10^-5^ vessels/um^2^ in the setting of extensive interstitial fibrosis and tubular atrophy. The mean MVD for all tissue was 28 ± 21x10^-5^ vessels/um^2^ (mean ± SD). MVA for all tissue ranged from 45–168 um^2^ with a mean of 95 ± 31 um^2^ (mean ± SD). As shown in Fig 7, there were correlations among MVD and MVA in the cortex, medulla, and entire parenchyma taken as a whole. MVD correlated between all of the kidney compartments (r = 0.76 to 0.87, P all < 0.0001), and MVA also correlated between all of the kidney compartments (r = 0.71 to 0.87, P all < 0.0001). MVD and MVA correlated with collagen III IHC morphometry for all tissue (r = 0.45, P = 0.03, and r = 0.58, P = 0.03, respectively), for the cortex (r = 0.75, P < 0.0001, and 0.63, P < 0.0001, respectively), and weakly for the medulla (r = 0.27, P = not significant [0.21] and r = 0.45, P = 0.03, respectively). Additional correlations regarding this data are shown in S8 Fig and S5 Table.

### Creatinine Correlation

As mentioned previously, creatinine did not cluster tightly with any of the measures based on the clustering of regressions that correlate shown in Fig 2. However, regression analysis did seem to indicate some correlations of low strength; and some of these were statistically significant. Statistically significant correlations for creatinine included relationships with visual assessment of cortical, all tissue, and medullary tubular atrophy (r = 0.47, p = 0.001; r = 0.45, p = 0.001; and p = 0.38, p = 0.009, respectively). There was also a statistically significant relationship between creatinine and visual assessment of cortical and all tissue trichrome fibrosis (r = 0.40, p = 0.006; and r = 0.33, p = 0.03, respectively). One of the EPCM measures, the morphometric assessment of EPCM using the trichrome “red” staining, also showed a statistically significant correlation with creatinine (r = - 0.30, p = 0.046), demonstrating that as the EPCM went down, then the creatinine tended to increase. Regarding other morphometric methods, only collagen III IHC morphometry showed a correlation r value at a low strength for creatinine versus collagen III IHC morphometry fibrosis for all tissue fibrosis (r = -0.40) and for cortical fibrosis (r = -0.36); however, these relationships were not statistically significant, although the number of cases with both collagen III IHC and creatinine data available was limited (S1 Data).

## Discussion

Our study demonstrates an image analysis approach for the study of fibrosis, EPCM, microvascularity, and other anatomic relationships in the kidney. Interpretation of our data is rather complex in some respects. We used multivariate linear regression to observe correlations among measurement parameters in this study. Admittedly, linear regression can have drawbacks with regard to overinterpretation of statistically significant results. As mentioned previously, we interpreted the data according guidelines that have been suggested for the interpretation of correlation r values [30, 31]; however, we recognize that much of our data will need further verification with additional studies.

The correlations between cortex and medulla are likely the one of the most informative aspects of this study. Correlations were moderate to high between the cortex and medulla for all of the measures in this study (Table 2). For comparison, additional correlations for various measures between cortex and medulla are shown in S7 Table; and all correlations for this study are shown in S1 Data. Since cases were selected to include a representation of medulla and the majority of the tissue in the cases was comprised of medulla, it is not surprising that there is a correlation between “all tissue” and medulla. Therefore, the correlations between cortex and medulla are more meaningful in many respects than the correlations involving “all tissue”. Although biopsies of predominantly medulla are typically avoided due to concerns for complications [32–35], biopsies containing predominantly medulla do still occur; therefore, the relationships between cortex and medulla found in this study could have utility when “medulla only” biopsies are encountered.

In this study, trichrome and T-P image analysis correlated between the cortex, medulla, and entire tissue; and visual assessment also correlated between the compartments. This helps support the idea that the medulla responds to injury in a similar anatomic and physiologic manner to the cortex. Examples of cases with medullary fibrosis are shown in S9 Fig. Collagen III deposition correlated between compartments but did not show correlation with other interstitial fibrosis measures, showing that additional components are likely important in extracellular matrix composition.

Interstitial "fibrosis" is a nonspecific concept. Most of the techniques that we used to define interstitial are chromogenic. For example, Masson trichrome provides an overall assessment of extracellular matrix; but it does not specifically address the composition of the interstitium and the variable alterations that may be occurring to the interstitial components. Trichrome stains, such as the Masson trichrome used in our study, are typically mordanted with agents such as phosphomolybdic acid; and this staining process is typically thought to intensify interstitial components along with other components of the Masson trichrome such as the Biebrich scarlet and the methyl or aniline blue.[36–38] Collagen type III immunohistochemistry provides a measure of a discrete protein component; however, as we have seen in other studies and reviewed elsewhere, using stains such as collagen III immunohistochemistry may neglect other components that may be present in fibrosis.[1, 2, 39, 40]

This study also demonstrates relationships between diminishing cortical and medullary EPCM. Furthermore, expansion of OS and IS are both associated with reduced medullary EPCM. Thus, as epithelial cell elements are lost, reactive/fibrotic responses cause zonal expansion in the OS and IS regions, which are quite important zones of vascular and anatomic support for the renal parenchyma. A nephrectomy specimen with IS and OS delineated is shown in S10 Fig. Kriz et al have noted that the OS in the human is very thin.[4, 7, 41] Pannabecker, Rosen, et al have noted this as well and are now in the process of examining the human outer medulla in depth and have recently published studies on the human inner medulla.[42] It is anticipated that the conclusions of their studies will further indicate that the OS of the outer medulla is virtually nonexistent in the human, at least from a functional perspective and possibly even from an anatomic perspective. The exact reason for the discrepancy in the biopsies, in which a thin (approximately 2 mm) OS was demonstrated is not clear, but may relate to the fact that the base of the cortex is almost entirely medullary ray, so that the exact interface between cortex and medulla may be hard to define, particularly in biopsies. The cortex in this area is represented as small triangles with the tip at the cortical medullary junction, so that the proximal tubular mass may be mistaken for OS. That said, it is certainly possible that proximal tubular hypertrophy expands the minimal stripe present, although there is no correlation between the thickness of the OS and the extent of fibrosis in this study or noteworthy prior studies.[5, 9, 10, 43]

Renal medullary vascularity shows parallels with the renal medulla, the renal cortex, and the entire renal parenchyma as a whole with regard to parameters such as MVD and MVA. Using CD34 IHC to highlight the renal vasculature in this cohort, the MVD algorithm employed in this study was useful in both qualitatively and quantitatively highlighting these relationships between cortical and medullary MVD and MVA; however, there is wide heterogeneity, indicating that further study may be worthwhile in larger cohorts and a wider variety of diseases. In addition, correlations were observed with increasing MVD and MVA accompanied by increasing collagen III IHC deposition. This could seem to indicate that increased microvascularity occurs in tandem with extracellular matrix deposition; however, there could also be some factors limiting the MVD algorithm in our cohort. We used CD34 with the MVD because it was readily available and robust; however, it is acknowledged that CD34 can stain interstitium in addition to vasculature, particularly in the setting of kidney injury [44–46]. Furthermore, we examined the vascular wall thickness measurement parameter provided by the microvessel analysis algorithm, and we found that these measurements also correlate with collagen III immunohistochemistry measurements, as shown in S11 Fig. This suggests that there may be an extracellular matrix expansion associated with the thickening of the vascular walls. Thus, staining of extracellular matrix and interstitium by CD34 could be confounding analysis by our CD34 algorithm. Additional markers of the renal vasculature could be more sensitive and specific for the vasculature and help further delineate different anatomic portions of the vascular bed; and MVD algorithm refinements will likely help quantitate the vasculature more accurately and further tease out important differences in portions of the vascular bed.

The measures in this study did not show tight correlations with renal function, as measured by creatinine; however, there are a number of factors that may have limited our ability to find correlation with renal function. The retrospective nature of this study made it difficult for us to obtain creatinine on a number of patients since many of the cases were consultative cases from outside hospitals; and since a number of institutions were involved, the creatinine data may not be entirely consistent. It is interesting that most of the significant correlations were seen with visual assessment of tubular atrophy and interstitial fibrosis. Weak trends were seen for the trichrome red EPCM assessment and collagen III IHC morphometry, although data was limited and not statistically significant for collagen III IHC. Future studies incorporating more accurate determinations of renal function than creatinine may be useful in disclosing correlations that this study was underpowered to detect.

Overall, the primary finding of this study was a proof of principle in some respects that the renal medulla shows tight relationships with the renal cortex. Renal medullary measurements may show utility in larger studies prospectively correlating medullary interstitial fibrosis with renal function in both human and animal models; and medullary measurements could prove useful in drug studies, clinical trials, and evaluation of biopsies containing only medulla. It is important to note, in this regard, that the correlation of fibrosis between the cortex and medulla does not necessarily mean, as most have supposed, the primary injury is in the cortex;[12] and indeed, studies such as this may provide a novel perspective to examine human chronic kidney disease through analysis of the renal medulla. In this manner, analyzing and quantitating medullary injury may provide a unique perspective to examine human chronic kidney disease.

## Supporting Information

S1 Data Correlation r values. (XLSX)

S1 Fig A two-way hierarchical clustering of key measured variables is shown. Cases are depicted along the y-axis, and measured features are displayed along the x-axis with related features being closer together.
(TIFF)

S2 Fig Correlation of different fibrosis measures is shown for the all of the tissue, the cortex (Ctx), and the medulla (Med) using image analysis of trichrome (Tri), trichrome analysis minus PAS analysis (T-P), and visual assessment (Vis). Regression lines and r values of corresponding measurements show how measurements correlate. Curved lines bound a density ellipse containing 95% of the measurements obtained.
(TIFF)

S3 Fig Correlation of assessment of tubular atrophy based on visual examination of the periodic acid–Schiff (PAS) stain is shown for the all of the tissue, the cortex (Ctx), and the medulla (Med). Regression lines and r values of corresponding measurements show how measurements correlate. Curved lines bound a density ellipse containing 95% of the measurements obtained.
(TIFF)

S4 Fig Correlation of assessment of tubular atrophy (TA) based on (A) trichrome morphometry and (B) visual examination of trichrome-stained slides. Measurements are performed for all of the tissue, the cortex (Ctx), and the medulla (Med). Regression lines and r values of corresponding measurements show how measurements correlate. Curved lines bound a density ellipse containing 95% of the measurements obtained.
(TIFF)

S5 Fig Correlation of fibrosis measures using collagen III immunohistochemistry (Col) is shown for the all of the tissue, the cortex (Ctx), and the medulla (Med). Regression lines and r values of corresponding measurements show how measurements correlate. Curved lines bound a density ellipse containing 95% of the measurements obtained.
(TIFF)

S6 Fig Epithelial cell mass measure correlations. (A) Correlation of epithelial cell mass measures is shown for the all of the tissue, the cortex (Ctx), and the medulla (Med). Regression lines and r values of corresponding measurements show how measurements correlate. Curved lines bound a density ellipse containing 95% of the measurements obtained. (B) Correlation of epithelial cell mass measures are shown. Measurements included the “Red” of the trichrome (RedTri), fibrosis using trichrome staining quantitation (denoted”-Tri”), and visual assessment of fibrosis (denoted “Vis-”). Measurements are performed on the all of the tissue, the cortex (Ctx), and the medulla (Med). Regression lines and r values of corresponding measurements show how measurements correlate. Curved lines bound a density ellipse containing 95% of the measurements obtained.
(TIFF)

S7 Fig (A) Correlation of outer and inner stripe width in millimeters (Out-Stri-mm and Inn-Stri-mm) with fibrosis measures, including all of the tissue, the cortex (Ctx), and the medulla (Med) using image analysis of trichrome (Tri). (B) Correlation of outer and inner stripe width in millimeters (Out-Stri-mm and Inn-Stri-mm) with measures of % epithelial mass by visual assessment, including all of the tissue, the cortex (Ctx). For both graphs, curved lines bound a density ellipse containing 95% of the measurements obtained.
(TIFF)

S8 Fig Microvessel correlations. (A) Correlation of measures of microvessel density (MVD in in vessels/um^2^) and mean vessel area (MVA in um^2^) are shown for the all of the tissue, the cortex (Ctx), and the medulla (Med). Regression lines and r values of corresponding measurements show how measurements correlate. Curved lines bound a density ellipse containing 95% of the measurements obtained. (B) Correlation of measures of microvessel density (MVD in in vessels/um^2^) and mean vessel area (MVA in um^2^) are shown for the all of the tissue, the cortex (Ctx), and the medulla (Med). In addition, correlations of MVD and MVA with collagen III immunohistochemistry morphometry are shown. Regression lines and r values of corresponding measurements show how measurements correlate. Curved lines bound a density ellipse containing 95% of the measurements obtained.
(TIFF)

S9 Fig Examples of cases with higher levels of fibrosis in the renal medulla with trichrome on the left and collagen III immunohistochemistry on the right (original magnification 200x). In the upper image, fibrosis occurs in a diffusely distributed manner; and the arrow denotes an atrophic tubule with a thickened basement membrane. In the lower images, the arrows denote a localized area of fibrosis.
(TIFF)

S10 Fig One of the nephrectomy specimen sections is shown with the outer stripe (OS) and inner stripe (IS) of the outer medulla delineated at 5x original magnification (trichrome). The lower images are higher magnification, showing the IS and OS (both 40x original magnification). The OS is composed of thick portions (limbs), containing tubules with more copious cytoplasm; whereas, the IS contains both thin as well as thick limbs.
(TIFF)

S11 Fig Correlations for measures of mean microvessel wall thickness (MVT in um) and collagen III immunohistochemistry morphometry are shown for the all of the tissue, the cortex (Ctx), and the medulla (Med). Regression lines and r values of corresponding measurements show how measurements correlate. Curved lines bound a density ellipse containing 95% of the measurements obtained.
(TIFF)

S1 Table Regression r values for correlation of different fibrosis measures are shown. Measurements are performed for the all of the tissue, the cortex (Ctx), and the medulla (Med) using image analysis of trichrome (Tri), trichrome analysis minus PAS analysis (T-P), and visual assessment (Vis). The P values corresponding to the regressions are also shown. Regression plots corresponding to these r values are shown in S2 Fig.
(DOC)

S2 Table Regression r values for correlation of tubular atrophy (TA) with (A) trichrome morphometry and (B) visual examination of trichrome-stained slides. Measurements are performed for all of the tissue, the cortex (Ctx), and the medulla (Med). The corresponding P values are also shown. Regression plots corresponding to these r values are shown in S4 Fig.
(DOC)

S3 Table Regression r values for correlation of epithelial cell mass [EPCM, Epithel below] measurements are shown. Measurements including the “Red” of the trichrome (RedTri) and cytokeratin (CK) immunohistochemistry were performed on the all of the tissue, the cortex (Ctx), and the medulla (Med). In addition, a visual assessment (Vis) assessment of EPCM was performed on the trichrome. Regression plots corresponding to these r values are shown in S6 Fig.
(DOC)

S4 Table (A) Correlation (r) values are given for the outer and inner stripe width in millimeters (Out-Stri-mm and Inn-Stri-mm) with fibrosis measures, including all of the tissue, the cortex (Ctx), and the medulla (Med) using image analysis of trichrome (Tri). (B) Correlation (r) values are given for the outer and inner stripe width in millimeters (Out-Stri-mm and Inn-Stri-mm) with measures of % epithelial mass by visual assessment, including all of the tissue, the cortex (Ctx). The corresponding P values are also shown.
(DOC)

S5 Table Correlation (r) values for measures of microvessel density (MVD in in vessels/um^2^) and mean vessel area (MVA in um^2^) are shown for the all of the tissue, the cortex (Ctx), and the medulla (Med). The corresponding P values are also shown.
(DOC)

S6 Table Patient demographics, clinical features, and selected biopsy findings are shown. (DOC)

S7 Table Correlation r values are shown in order of strength of association for the measurements that had “very high” and “high” correlations between cortex and medulla^. (DOC)
