# Supplementary figures and images for: Renal Medullary and Cortical Correlates in Fibrosis, Epithelial Mass, Microvascularity, and Microanatomy Using Whole Slide Image Analysis Morphometry
Source: PLoS One. 2016 Aug 30;11(8):e0161019. doi: 10.1371/journal.pone.0161019 (PMC5004931; doi:10.1371/journal.pone.0161019)

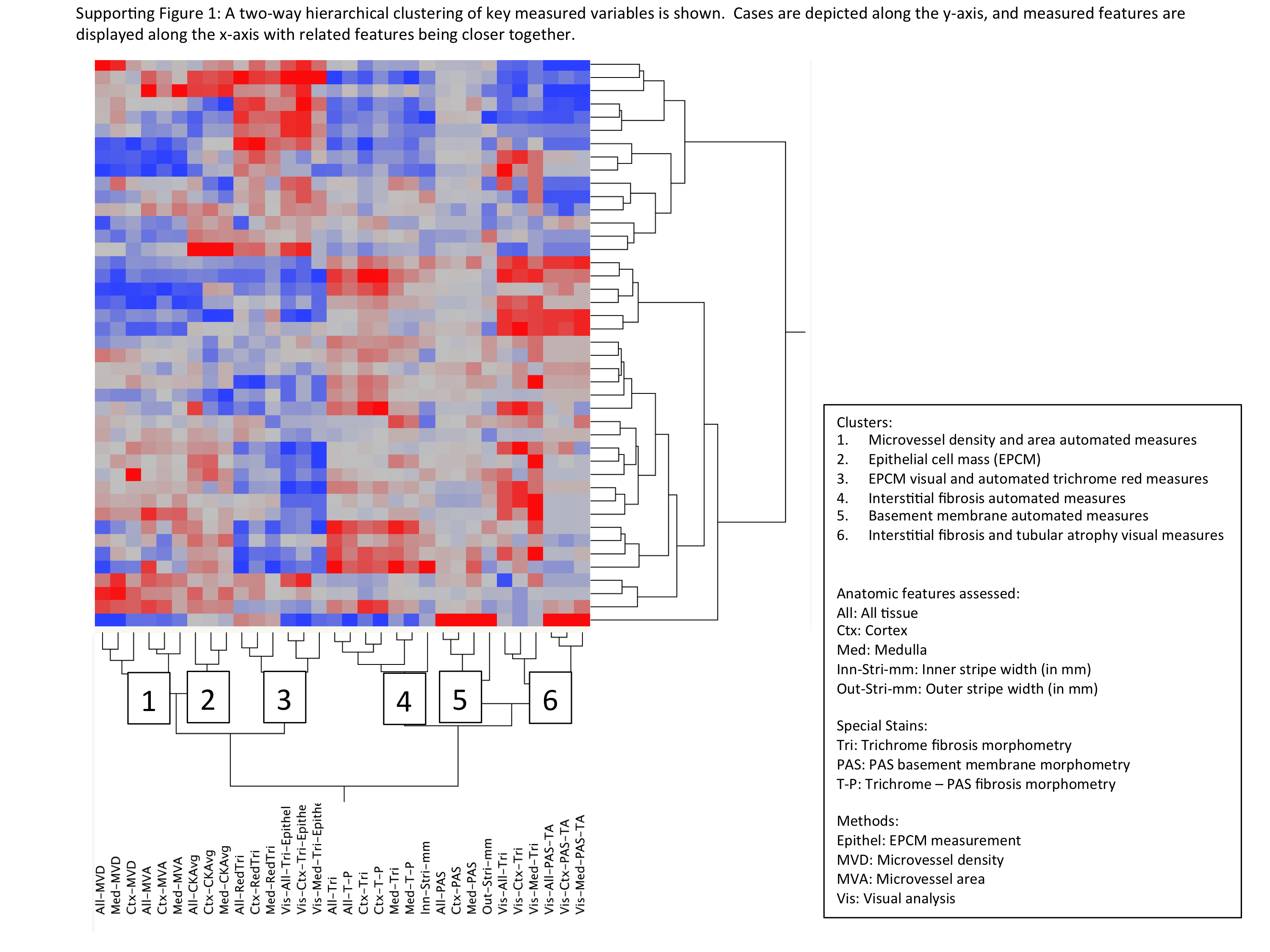

Supplement: S1 Fig — Cases are depicted along the y-axis, and measured features are displayed along the x-axis with related features being closer together. (TIFF) [file pone.0161019.s002.tiff]

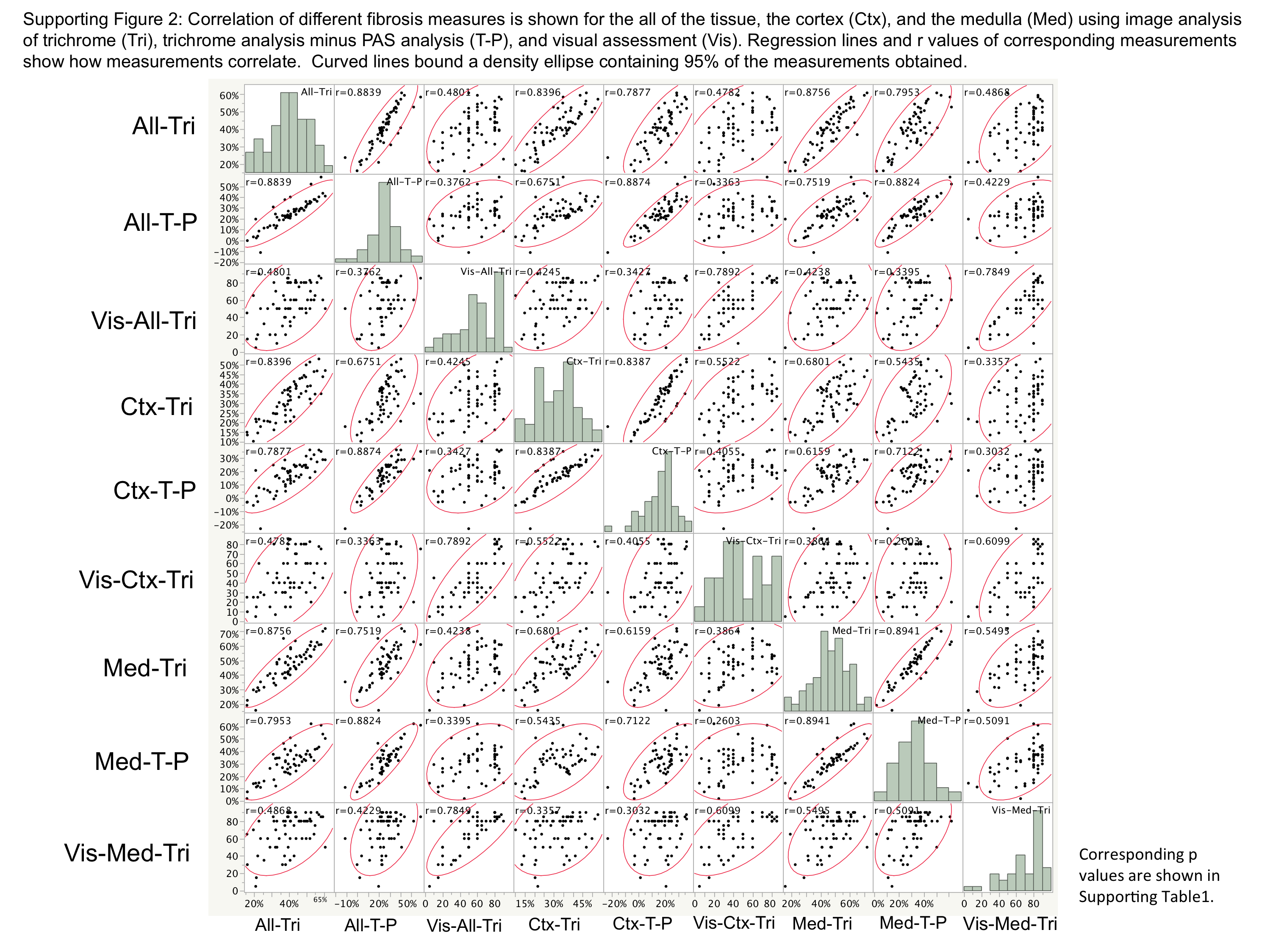

Supplement: S2 Fig — Regression lines and r values of corresponding measurements show how measurements correlate. Curved lines bound a density ellipse containing 95% of the measurements obtained. (TIFF) [file pone.0161019.s003.tiff]

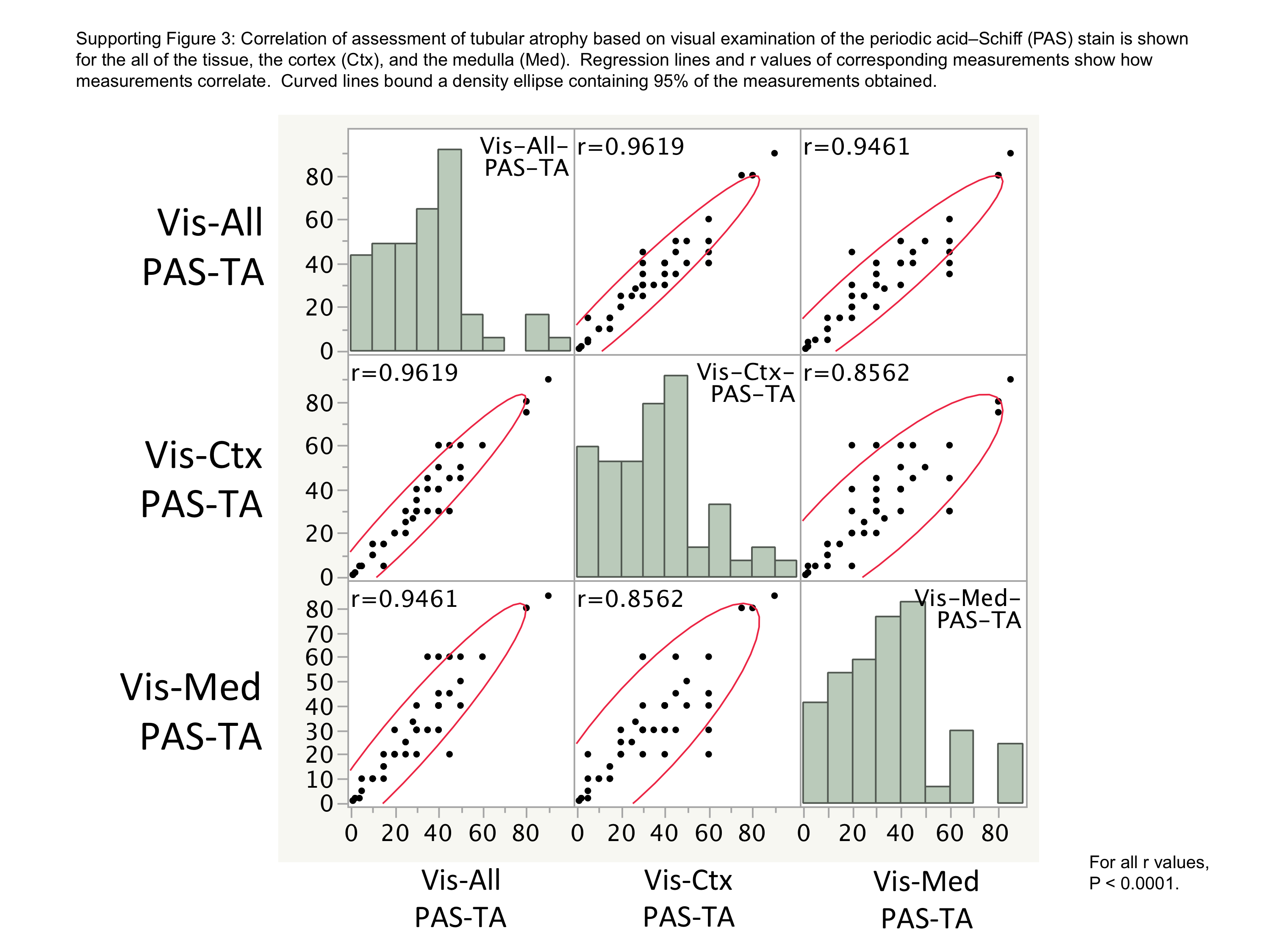

Supplement: S3 Fig — Regression lines and r values of corresponding measurements show how measurements correlate. Curved lines bound a density ellipse containing 95% of the measurements obtained. (TIFF) [file pone.0161019.s004.tiff]

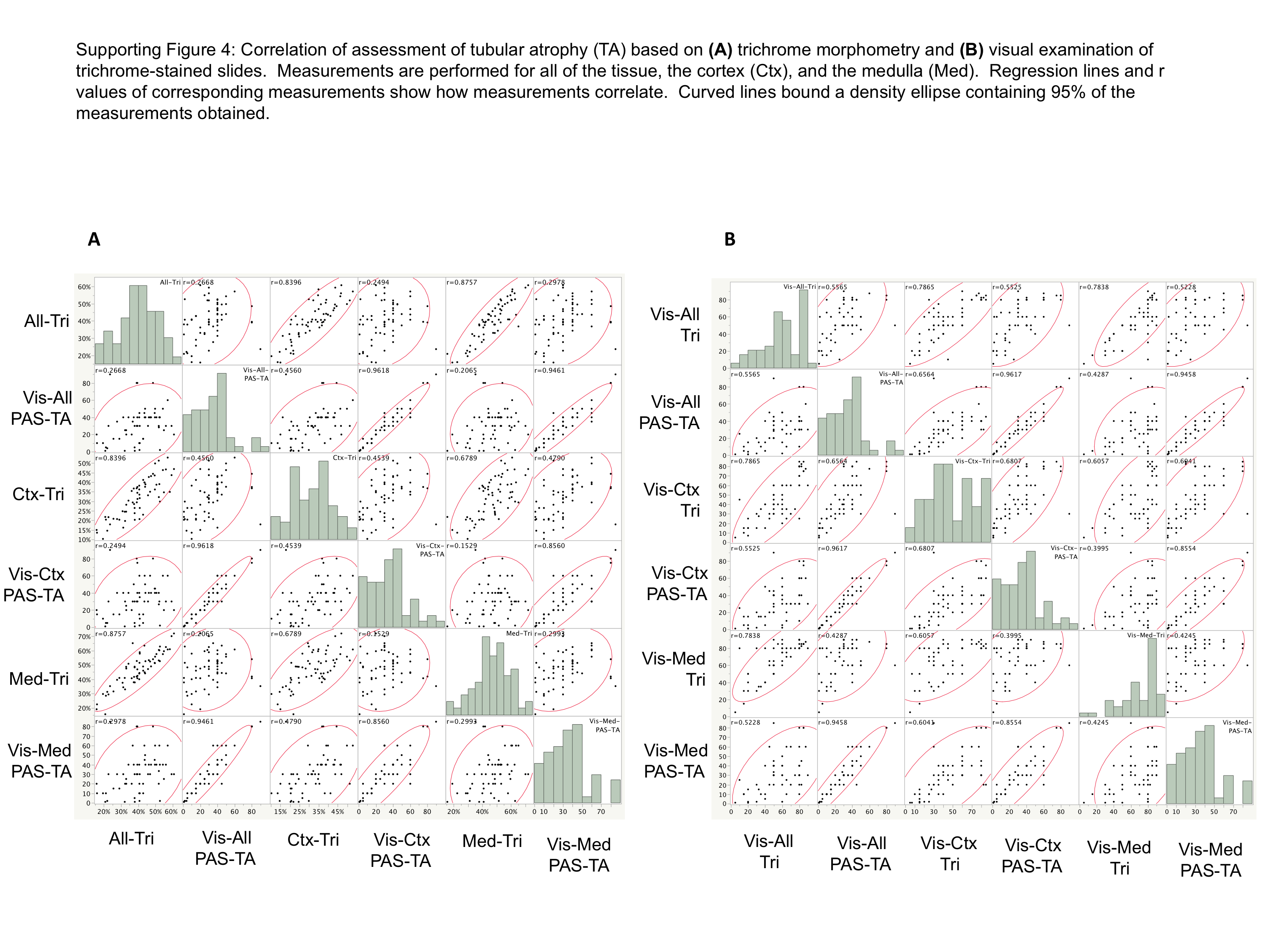

Supplement: S4 Fig — Correlation of assessment of tubular atrophy (TA) based on (A) trichrome morphometry and (B) visual examination of trichrome-stained slides. Measurements are performed for all of the tissue, the cortex (Ctx), and the medulla (Med). Regression lines and r values of corresponding measurements show how measurements correlate. Curved lines bound a density ellipse containing 95% of the measurements obtained. (TIFF) [file pone.0161019.s005.tiff]

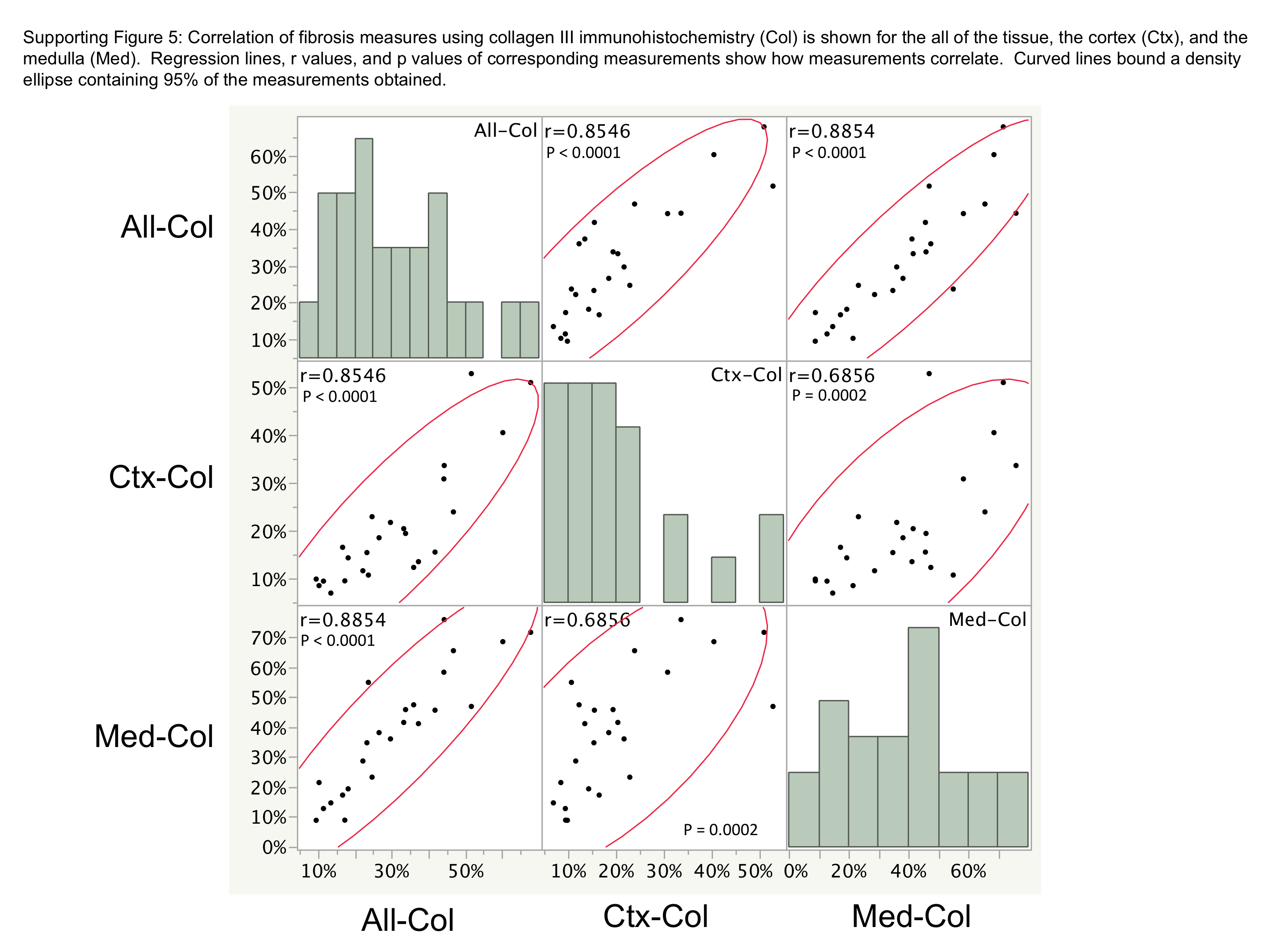

Supplement: S5 Fig — Regression lines and r values of corresponding measurements show how measurements correlate. Curved lines bound a density ellipse containing 95% of the measurements obtained. (TIFF) [file pone.0161019.s006.tiff]

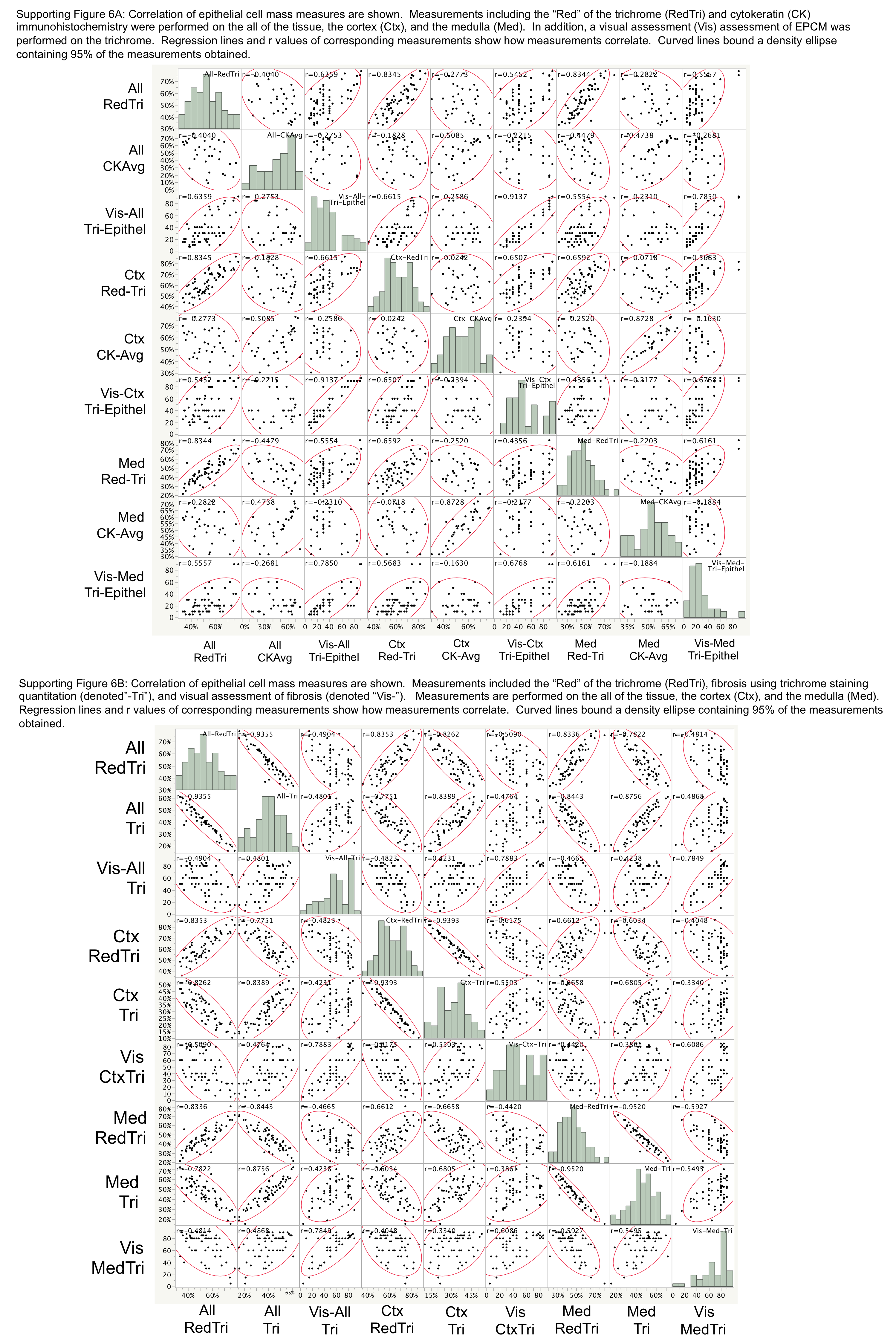

Supplement: S6 Fig — (A) Correlation of epithelial cell mass measures is shown for the all of the tissue, the cortex (Ctx), and the medulla (Med). Regression lines and r values of corresponding measurements show how measurements correlate. Curved lines bound a density ellipse containing 95% of the measurements obtained. (B) Correlation of epithelial cell mass measures are shown. Measurements included the “Red” of the trichrome (RedTri), fibrosis using trichrome staining quantitation (denoted”-Tri”), and visual assessment of fibrosis (denoted “Vis-”). Measurements are performed on the all of the tissue, the cortex (Ctx), and the medulla (Med). Regression lines and r values of corresponding measurements show how measurements correlate. Curved lines bound a density ellipse containing 95% of the measurements obtained. (TIFF) [file pone.0161019.s007.tiff]

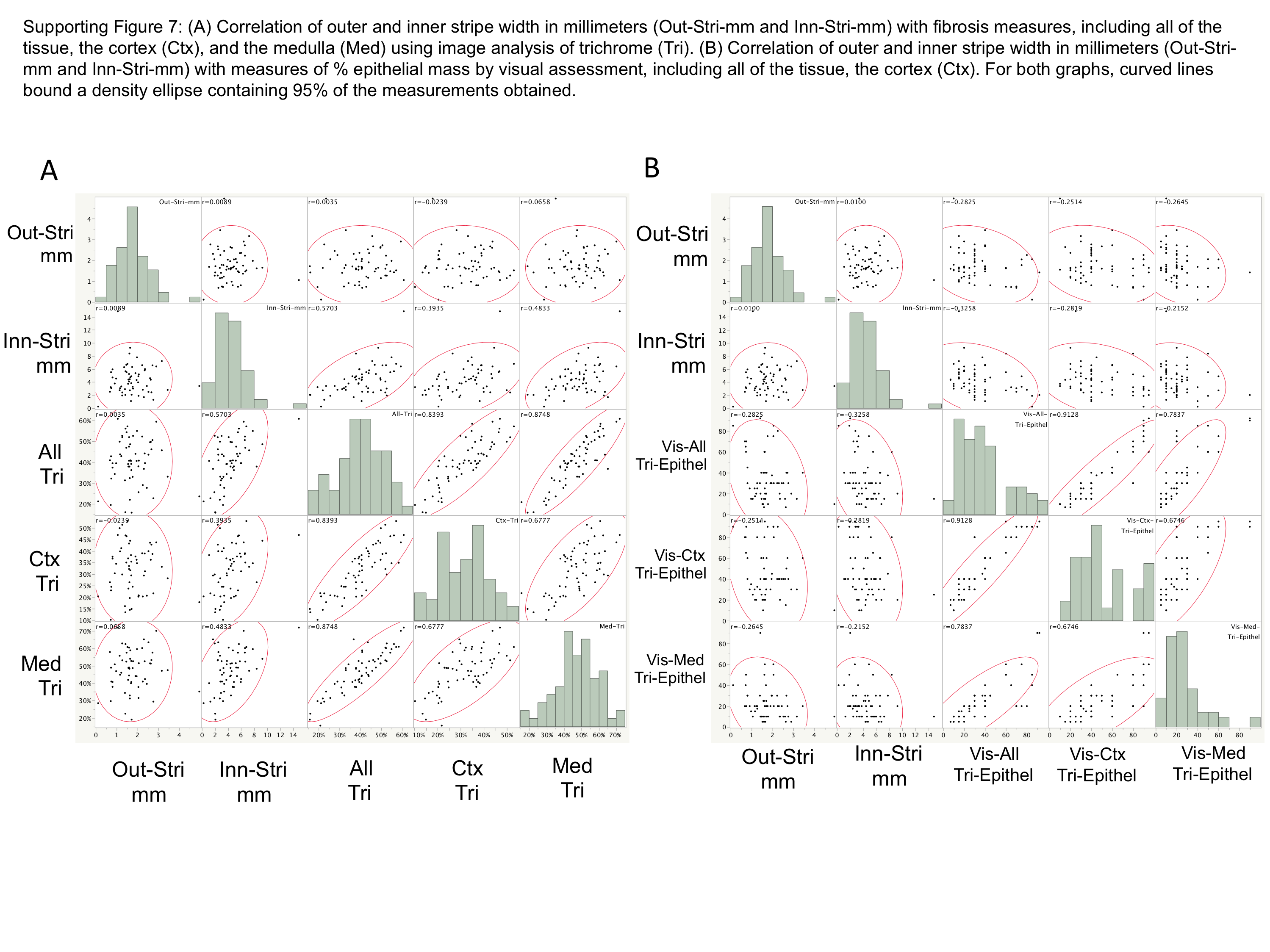

Supplement: S7 Fig — (A) Correlation of outer and inner stripe width in millimeters (Out-Stri-mm and Inn-Stri-mm) with fibrosis measures, including all of the tissue, the cortex (Ctx), and the medulla (Med) using image analysis of trichrome (Tri). (B) Correlation of outer and inner stripe width in millimeters (Out-Stri-mm and Inn-Stri-mm) with measures of % epithelial mass by visual assessment, including all of the tissue, the cortex (Ctx). For both graphs, curved lines bound a density ellipse containing 95% of the measurements obtained. (TIFF) [file pone.0161019.s008.tiff]

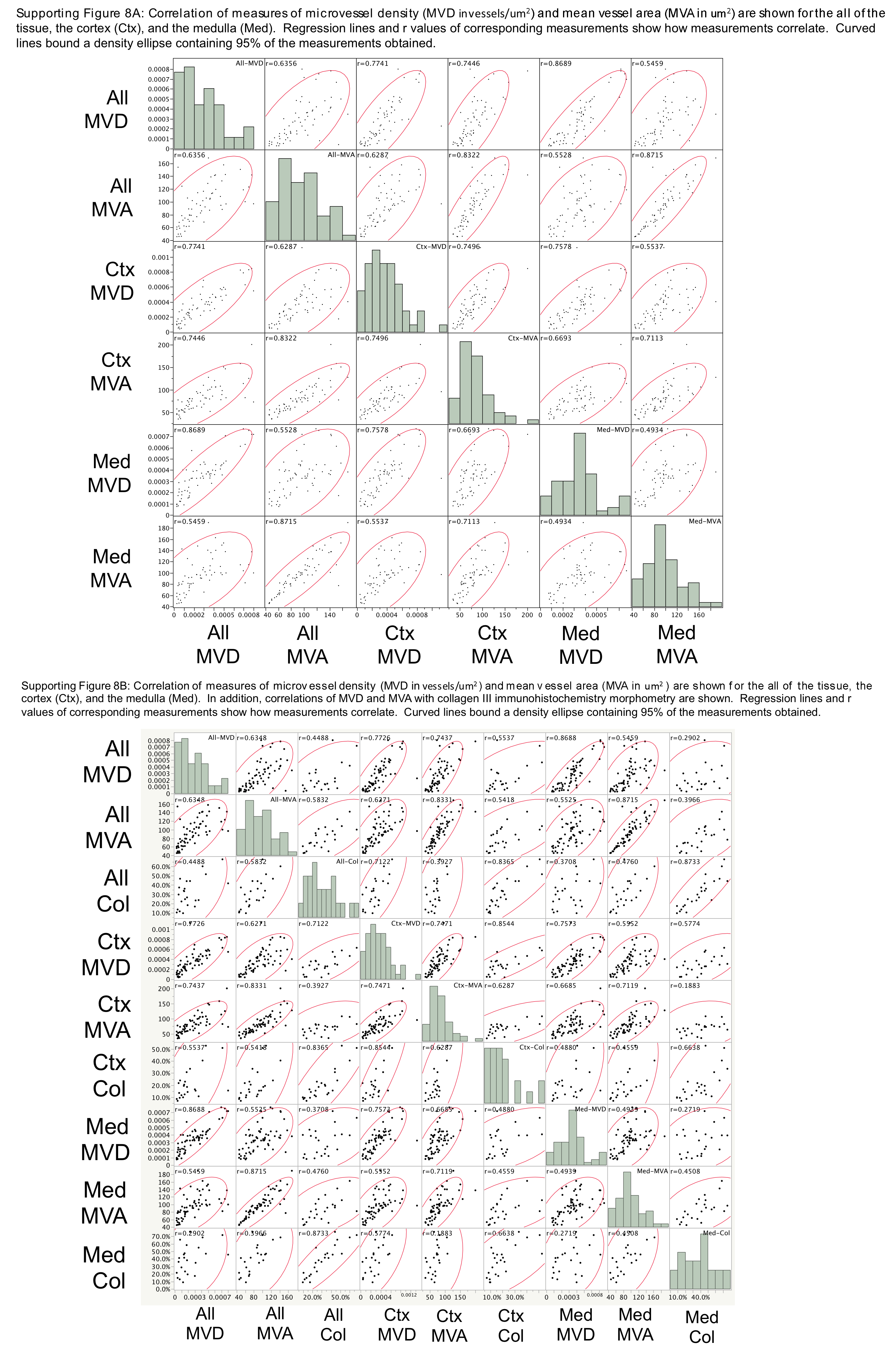

Supplement: S8 Fig — (A) Correlation of measures of microvessel density (MVD in in vessels/um2) and mean vessel area (MVA in um2) are shown for the all of the tissue, the cortex (Ctx), and the medulla (Med). Regression lines and r values of corresponding measurements show how measurements correlate. Curved lines bound a density ellipse containing 95% of the measurements obtained. (B) Correlation of measures of microvessel density (MVD in in vessels/um2) and mean vessel area (MVA in um2) are shown for the all of the tissue, the cortex (Ctx), and the medulla (Med). In addition, correlations of MVD and MVA with collagen III immunohistochemistry morphometry are shown. Regression lines and r values of corresponding measurements show how measurements correlate. Curved lines bound a density ellipse containing 95% of the measurements obtained. (TIFF) [file pone.0161019.s009.tiff]

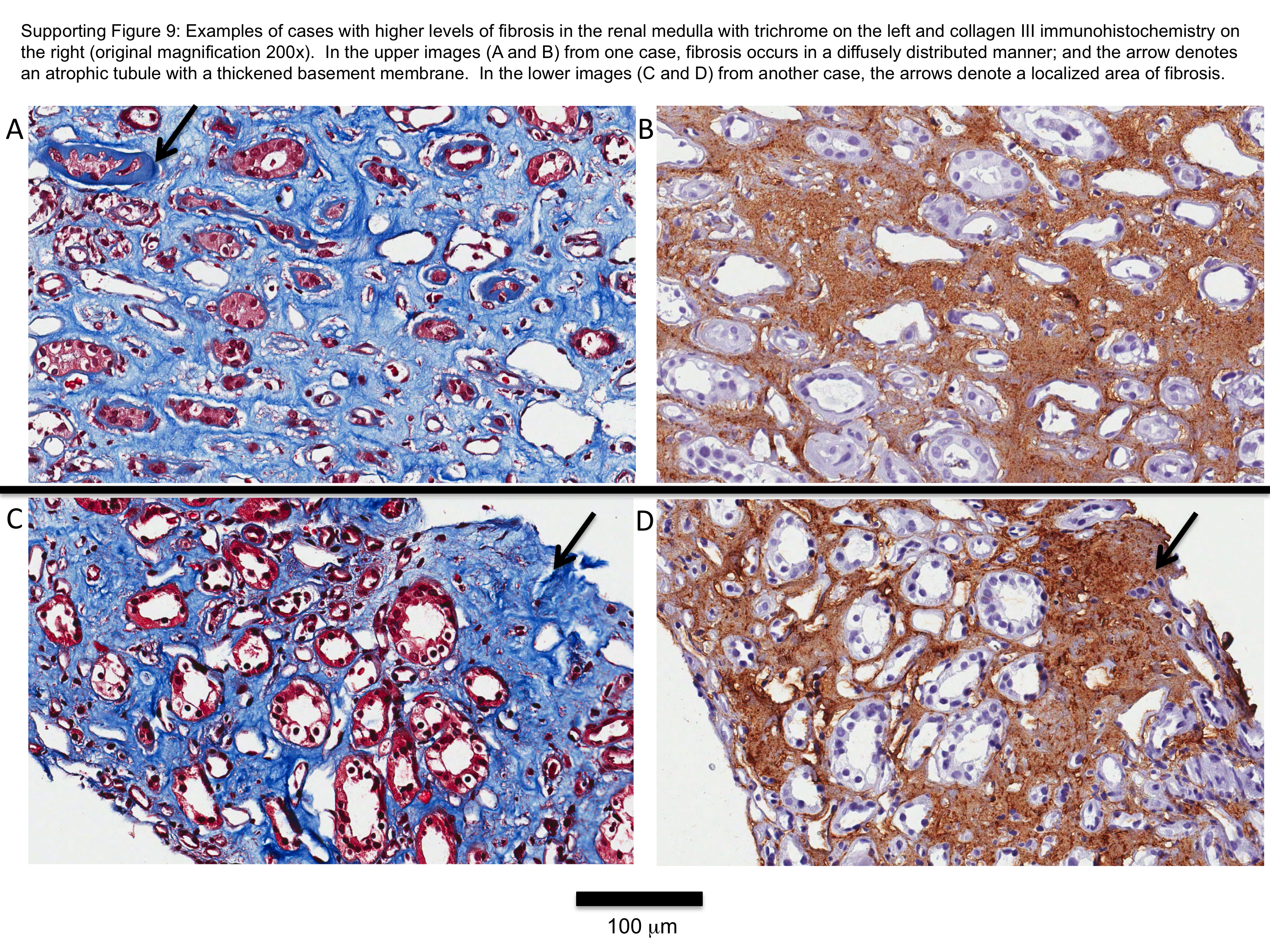

Supplement: S9 Fig — In the upper image, fibrosis occurs in a diffusely distributed manner; and the arrow denotes an atrophic tubule with a thickened basement membrane. In the lower images, the arrows denote a localized area of fibrosis. (TIFF) [file pone.0161019.s010.tiff]

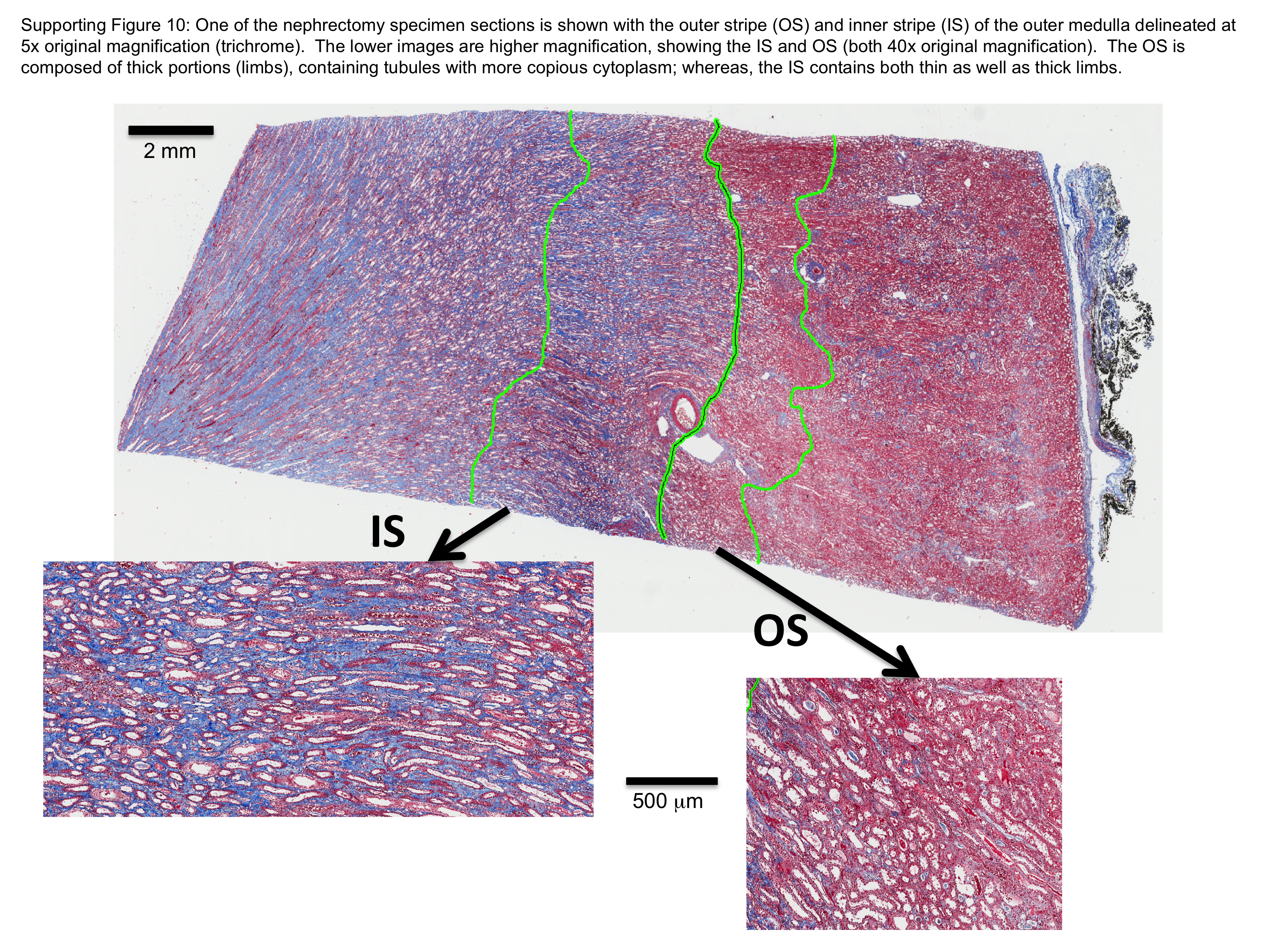

Supplement: S10 Fig — The lower images are higher magnification, showing the IS and OS (both 40x original magnification). The OS is composed of thick portions (limbs), containing tubules with more copious cytoplasm; whereas, the IS contains both thin as well as thick limbs. (TIFF) [file pone.0161019.s011.tiff]

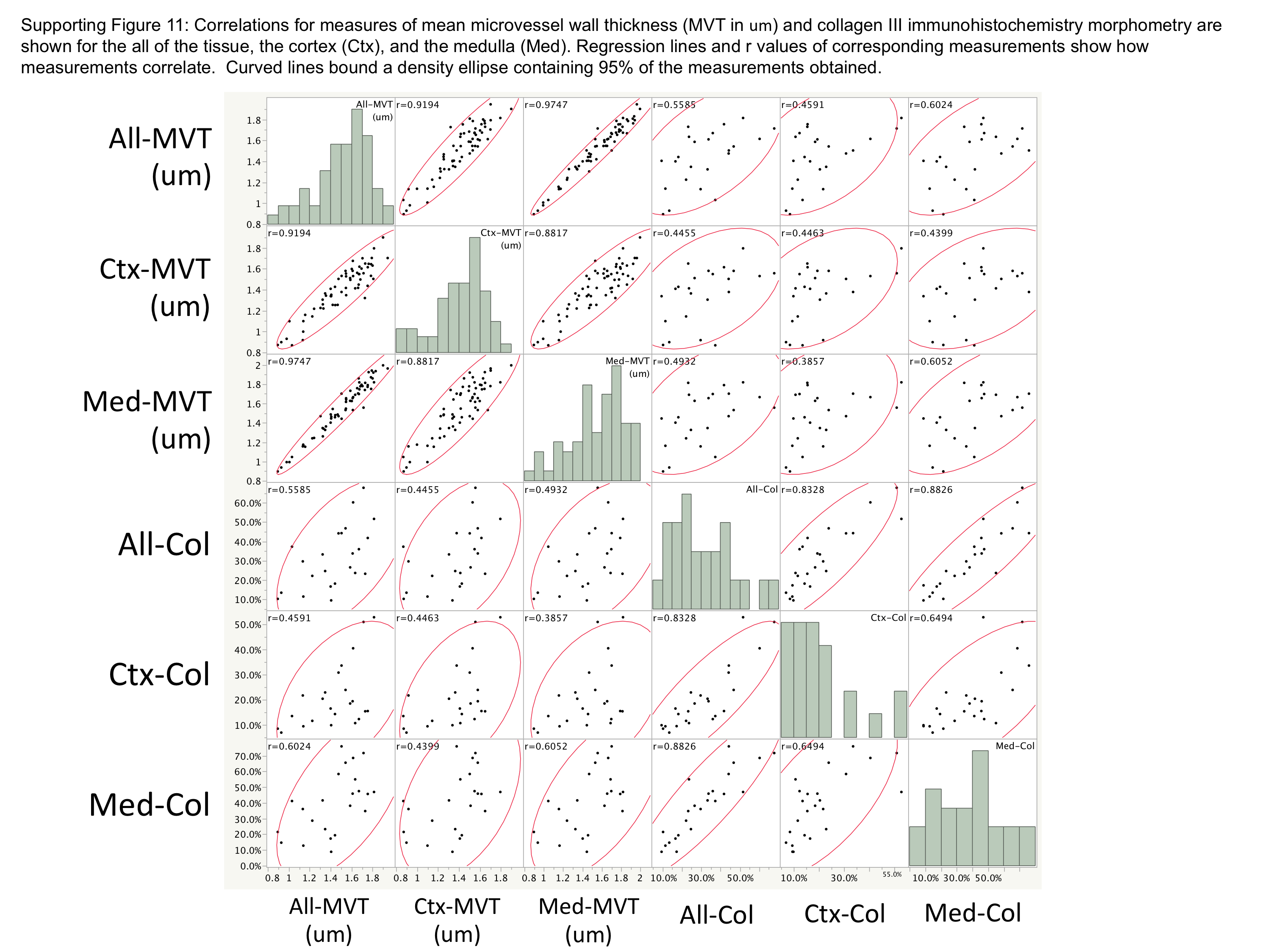

Supplement: S11 Fig — Regression lines and r values of corresponding measurements show how measurements correlate. Curved lines bound a density ellipse containing 95% of the measurements obtained. (TIFF) [file pone.0161019.s012.tiff]
