# Supplementary material for: Renal Medullary and Cortical Correlates in Fibrosis, Epithelial Mass, Microvascularity, and Microanatomy Using Whole Slide Image Analysis Morphometry
Source: PLoS One. 2016 Aug 30;11(8):e0161019. doi: 10.1371/journal.pone.0161019 (PMC5004931; doi:10.1371/journal.pone.0161019)
Supplement: S1 Table — Measurements are performed for the all of the tissue, the cortex (Ctx), and the medulla (Med) using image analysis of trichrome (Tri), trichrome analysis minus PAS analysis (T-P), and visual assessment (Vis). The P values corresponding to the regressions are also shown. Regression plots corresponding to these r values are shown in S2 Fig. (DOC) [file pone.0161019.s013.doc]

**Supporting Table 1:** Regression r values for correlation of different fibrosis measures are shown. Measurements are performed for the all of the tissue, the cortex (Ctx), and the medulla (Med) using image analysis of trichrome (Tri), trichrome analysis minus PAS analysis (T-P), and visual assessment (Vis). The P values corresponding to the regressions are also shown. Regression plots corresponding to these r values are shown in Supporting Figure 1.

| **Regression r Values:** | | | | | | | | | |
| --- | --- | --- | --- | --- | --- | --- | --- | --- | --- |
|  |  |  |  |  |  |  |  |  |  |
|  | **All-Tri** | **All-T-P** | **Vis-All-Tri** | **Ctx-Tri** | **Ctx-T-P** | **Vis-Ctx-Tri** | **Med-Tri** | **Med-T-P** | **Vis-Med-Tri** |
| All-Tri | 1.00 | 0.88 | 0.48 | 0.84 | 0.79 | 0.48 | 0.88 | 0.80 | 0.49 |
| All-T-P | 0.88 | 1.00 | 0.38 | 0.68 | 0.89 | 0.34 | 0.75 | 0.88 | 0.42 |
| Vis-All-Tri | 0.48 | 0.38 | 1.00 | 0.42 | 0.34 | 0.79 | 0.42 | 0.34 | 0.78 |
| Ctx-Tri | 0.84 | 0.68 | 0.42 | 1.00 | 0.84 | 0.55 | 0.68 | 0.54 | 0.34 |
| Ctx-T-P | 0.79 | 0.89 | 0.34 | 0.84 | 1.00 | 0.41 | 0.62 | 0.71 | 0.30 |
| Vis-Ctx-Tri | 0.48 | 0.34 | 0.79 | 0.55 | 0.41 | 1.00 | 0.39 | 0.26 | 0.61 |
| Med-Tri | 0.88 | 0.75 | 0.42 | 0.68 | 0.62 | 0.39 | 1.00 | 0.89 | 0.55 |
| Med-T-P | 0.80 | 0.88 | 0.34 | 0.54 | 0.71 | 0.26 | 0.89 | 1.00 | 0.51 |
| Vis-Med-Tri | 0.49 | 0.42 | 0.78 | 0.34 | 0.30 | 0.61 | 0.55 | 0.51 | 1.00 |
|  |  |  |  |  |  |  |  |  |  |
| **Corresponding Regression P values:** | | | | | | | | | |
|  |  |  |  |  |  |  |  |  |  |
|  | **All-Tri** | **All-T-P** | **Vis-All-Tri** | **Ctx-Tri** | **Ctx-T-P** | **Vis-Ctx-Tri** | **Med-Tri** | **Med-T-P** | **Vis-Med-Tri** |
| All-Tri | <0.00001 | <0.00001 | 0.00007 | <0.00001 | <0.00001 | 0.00008 | <0.00001 | <0.00001 | 0.00005 |
| All-T-P | <0.00001 | <0.00001 | 0.00305 | <0.00001 | <0.00001 | 0.00861 | <0.00001 | <0.00001 | 0.00076 |
| Vis-All-Tri | 0.00007 | 0.00305 | <0.00001 | 0.00059 | 0.00735 | <0.00001 | 0.00054 | 0.00912 | <0.00001 |
| Ctx-Tri | <0.00001 | <0.00001 | 0.00059 | <0.00001 | <0.00001 | <0.00001 | <0.00001 | 0.00001 | 0.00764 |
| Ctx-T-P | <0.00001 | <0.00001 | 0.00735 | <0.00001 | <0.00001 | 0.00131 | <0.00001 | <0.00001 | 0.01851 |
| Vis-Ctx-Tri | 0.00008 | 0.00861 | <0.00001 | <0.00001 | 0.00131 | <0.00001 | 0.00192 | 0.04844 | <0.00001 |
| Med-Tri | <0.00001 | <0.00001 | 0.00054 | <0.00001 | <0.00001 | 0.00192 | <0.00001 | <0.00001 | <0.00001 |
| Med-T-P | <0.00001 | <0.00001 | 0.00912 | 0.00001 | <0.00001 | 0.04844 | <0.00001 | <0.00001 | 0.00004 |
| Vis-Med-Tri | 0.00005 | 0.00076 | <0.00001 | 0.00764 | 0.01851 | <0.00001 | <0.00001 | 0.00004 | <0.00001 |
