# Supplementary material for: Renal Medullary and Cortical Correlates in Fibrosis, Epithelial Mass, Microvascularity, and Microanatomy Using Whole Slide Image Analysis Morphometry
Source: PLoS One. 2016 Aug 30;11(8):e0161019. doi: 10.1371/journal.pone.0161019 (PMC5004931; doi:10.1371/journal.pone.0161019)
Supplement: S2 Table — Regression r values for correlation of tubular atrophy (TA) with (A) trichrome morphometry and (B) visual examination of trichrome-stained slides. Measurements are performed for all of the tissue, the cortex (Ctx), and the medulla (Med). The corresponding P values are also shown. Regression plots corresponding to these r values are shown in S4 Fig. (DOC) [file pone.0161019.s014.doc]

**Supporting Table 2:** Regression r values for correlation of tubular atrophy (TA) with (A) trichrome morphometry and (B) visual examination of trichrome-stained slides. Measurements are performed for all of the tissue, the cortex (Ctx), and the medulla (Med). The corresponding P values are also shown. Regression plots corresponding to these r values are shown in Supporting Figure 3.

| **A. Regression r values** | | | | | | |
| --- | --- | --- | --- | --- | --- | --- |
|  |  |  |  |  |  |  |
|  | **All-Tri** | **Vis-All-PAS-TA** | **Ctx-Tri** | **Vis-Ctx-PAS-TA** | **Med-Tri** | **Vis-Med-PAS-TA** |
| All-Tri | 1.00 | 0.27 | 0.84 | 0.25 | 0.88 | 0.30 |
| Vis-All-PAS-TA | 0.27 | 1.00 | 0.46 | 0.96 | 0.21 | 0.95 |
| Ctx-Tri | 0.84 | 0.46 | 1.00 | 0.45 | 0.68 | 0.48 |
| Vis-Ctx-PAS-TA | 0.25 | 0.96 | 0.45 | 1.00 | 0.15 | 0.86 |
| Med-Tri | 0.88 | 0.21 | 0.68 | 0.15 | 1.00 | 0.30 |
| Vis-Med-PAS-TA | 0.30 | 0.95 | 0.48 | 0.86 | 0.30 | 1.00 |
|  |  |  |  |  |  |  |
| **A. Corresponding P values** | | | | | | |
|  |  |  |  |  |  |  |
|  | **All-Tri** | **Vis-All-PAS-TA** | **Ctx-Tri** | **Vis-Ctx-PAS-TA** | **Med-Tri** | **Vis-Med-PAS-TA** |
| All-Tri | <0.00001 | 0.03452 | <0.00001 | 0.05064 | <0.00001 | 0.01775 |
| Vis-All-PAS-TA | 0.03452 | <0.00001 | 0.00020 | <0.00001 | 0.10440 | <0.00001 |
| Ctx-Tri | <0.00001 | 0.00020 | <0.00001 | 0.00021 | <0.00001 | 0.00008 |
| Vis-Ctx-PAS-TA | 0.05064 | <0.00001 | 0.00021 | <0.00001 | 0.23558 | <0.00001 |
| Med-Tri | <0.00001 | 0.10440 | <0.00001 | 0.23558 | <0.00001 | 0.01716 |
| Vis-Med-PAS-TA | 0.01775 | <0.00001 | 0.00008 | <0.00001 | 0.01716 | <0.00001 |
|  |  |  |  |  |  |  |
|  |  |  |  |  |  |  |
| **B. Regression r values** | | | | | | |
|  |  |  |  |  |  |  |
|  | **Vis-All-Tri** | **Vis-All-PAS-TA** | **Vis-Ctx-Tri** | **Vis-Ctx-PAS-TA** | **Vis-Med-Tri** | **Vis-Med-PAS-TA** |
| Vis-All-Tri | 1.00 | 0.56 | 0.79 | 0.55 | 0.78 | 0.52 |
| Vis-All-PAS-TA | 0.56 | 1.00 | 0.66 | 0.96 | 0.43 | 0.95 |
| Vis-Ctx-Tri | 0.79 | 0.66 | 1.00 | 0.68 | 0.61 | 0.60 |
| Vis-Ctx-PAS-TA | 0.55 | 0.96 | 0.68 | 1.00 | 0.40 | 0.86 |
| Vis-Med-Tri | 0.78 | 0.43 | 0.61 | 0.40 | 1.00 | 0.42 |
| Vis-Med-PAS-TA | 0.52 | 0.95 | 0.60 | 0.86 | 0.42 | 1.00 |
|  |  |  |  |  |  |  |
| **B. Corresponding P values** | | | | | | |
|  |  |  |  |  |  |  |
|  | **Vis-All-Tri** | **Vis-All-PAS-TA** | **Vis-Ctx-Tri** | **Vis-Ctx-PAS-TA** | **Vis-Med-Tri** | **Vis-Med-PAS-TA** |
| Vis-All-Tri | <0.00001 | <0.00001 | <0.00001 | <0.00001 | <0.00001 | 0.00001 |
| Vis-All-PAS-TA | <0.00001 | <0.00001 | <0.00001 | <0.00001 | 0.00046 | <0.00001 |
| Vis-Ctx-Tri | <0.00001 | <0.00001 | <0.00001 | <0.00001 | <0.00001 | <0.00001 |
| Vis-Ctx-PAS-TA | <0.00001 | <0.00001 | <0.00001 | <0.00001 | 0.00130 | <0.00001 |
| Vis-Med-Tri | <0.00001 | 0.00046 | <0.00001 | 0.00130 | <0.00001 | 0.00053 |
| Vis-Med-PAS-TA | 0.00001 | <0.00001 | <0.00001 | <0.00001 | 0.00053 | <0.00001 |
