# Supplementary material for: Renal Medullary and Cortical Correlates in Fibrosis, Epithelial Mass, Microvascularity, and Microanatomy Using Whole Slide Image Analysis Morphometry
Source: PLoS One. 2016 Aug 30;11(8):e0161019. doi: 10.1371/journal.pone.0161019 (PMC5004931; doi:10.1371/journal.pone.0161019)
Supplement: S3 Table — Measurements including the “Red” of the trichrome (RedTri) and cytokeratin (CK) immunohistochemistry were performed on the all of the tissue, the cortex (Ctx), and the medulla (Med). In addition, a visual assessment (Vis) assessment of EPCM was performed on the trichrome. Regression plots corresponding to these r values are shown in S6 Fig. (DOC) [file pone.0161019.s015.doc]

**Supporting Table 3:** Regression r values for correlation of epithelial cell mass [EPCM, Epithel below] measurements are shown. Measurements including the “Red” of the trichrome (RedTri) and cytokeratin (CK) immunohistochemistry were performed on the all of the tissue, the cortex (Ctx), and the medulla (Med). In addition, a visual assessment (Vis) assessment of EPCM was performed on the trichrome. Regression plots corresponding to these r values are shown in Supporting Figure 5.

| **Regression r values** |
| --- |
| |  | **All-RedTri** | **All-CKAvg** | **Vis-All-Tri-Epithel** | **Ctx-RedTri** | **Ctx-CKAvg** | **Vis-Ctx-Tri-Epithel** | **Med-RedTri** | **Med-CKAvg** | **Vis-Med-Tri-Epithel** | | --- | --- | --- | --- | --- | --- | --- | --- | --- | --- | | All-RedTri | 1.00 | -0.40 | 0.64 | 0.83 | -0.28 | 0.55 | 0.83 | -0.28 | 0.56 | | All-CKAvg | -0.40 | 1.00 | -0.28 | -0.18 | 0.51 | -0.22 | -0.45 | 0.47 | -0.27 | | Vis-All-Tri-Epithel | 0.64 | -0.28 | 1.00 | 0.66 | -0.26 | 0.91 | 0.56 | -0.23 | 0.79 | | Ctx-RedTri | 0.83 | -0.18 | 0.66 | 1.00 | -0.02 | 0.65 | 0.66 | -0.07 | 0.57 | | Ctx-CKAvg | -0.28 | 0.51 | -0.26 | -0.02 | 1.00 | -0.24 | -0.25 | 0.87 | -0.16 | | Vis-Ctx-Tri-Epithel | 0.55 | -0.22 | 0.91 | 0.65 | -0.24 | 1.00 | 0.44 | -0.22 | 0.68 | | Med-RedTri | 0.83 | -0.45 | 0.56 | 0.66 | -0.25 | 0.44 | 1.00 | -0.22 | 0.62 | | Med-CKAvg | -0.28 | 0.47 | -0.23 | -0.07 | 0.87 | -0.22 | -0.22 | 1.00 | -0.19 | | Vis-Med-Tri-Epithel | 0.56 | -0.27 | 0.79 | 0.57 | -0.16 | 0.68 | 0.62 | -0.19 | 1.00 |   **Corresponding P values** |

|  | **All-RedTri** | **All-CKAvg** | **Vis-All-Tri-Epithel** | **Ctx-RedTri** | **Ctx-CKAvg** | **Vis-Ctx-Tri-Epithel** | **Med-RedTri** | **Med-CKAvg** | **Vis-Med-Tri-Epithel** |
| --- | --- | --- | --- | --- | --- | --- | --- | --- | --- |
| All-RedTri | 0.0178 | <.0001 | 0.1151 | 0.3008 | 0.0021 | 0.2081 | 0.0079 | 0.0046 | 0.1253 |
| All-CKAvg | <.0001 | 0.1151 | <.0001 | <.0001 | 0.1397 | <.0001 | <.0001 | 0.1888 | <.0001 |
| Vis-All-Tri-Epithel | <.0001 | 0.3008 | <.0001 | <.0001 | 0.8921 | <.0001 | <.0001 | 0.6865 | <.0001 |
| Ctx-RedTri | 0.1124 | 0.0021 | 0.1397 | 0.8921 | <.0001 | 0.1727 | 0.1505 | <.0001 | 0.3569 |
| Ctx-CKAvg | <.0001 | 0.2081 | <.0001 | <.0001 | 0.1727 | <.0001 | 0.0004 | 0.2162 | <.0001 |
| Vis-Ctx-Tri-Epithel | <.0001 | 0.0079 | <.0001 | <.0001 | 0.1505 | 0.0004 | <.0001 | 0.2106 | <.0001 |
| Med-RedTri | 0.1058 | 0.0046 | 0.1888 | 0.6865 | <.0001 | 0.2162 | 0.2106 | <.0001 | 0.2859 |
| Med-CKAvg | <.0001 | 0.1253 | <.0001 | <.0001 | 0.3569 | <.0001 | <.0001 | 0.2859 | <.0001 |
| Vis-Med-Tri-Epithel | 0.0178 | <.0001 | 0.1151 | 0.3008 | 0.0021 | 0.2081 | 0.0079 | 0.0046 | 0.1253 |
