# Supplementary material for: Renal Medullary and Cortical Correlates in Fibrosis, Epithelial Mass, Microvascularity, and Microanatomy Using Whole Slide Image Analysis Morphometry
Source: PLoS One. 2016 Aug 30;11(8):e0161019. doi: 10.1371/journal.pone.0161019 (PMC5004931; doi:10.1371/journal.pone.0161019)
Supplement: S4 Table — (A) Correlation (r) values are given for the outer and inner stripe width in millimeters (Out-Stri-mm and Inn-Stri-mm) with fibrosis measures, including all of the tissue, the cortex (Ctx), and the medulla (Med) using image analysis of trichrome (Tri). (B) Correlation (r) values are given for the outer and inner stripe width in millimeters (Out-Stri-mm and Inn-Stri-mm) with measures of % epithelial mass by visual assessment, including all of the tissue, the cortex (Ctx). The corresponding P values are also shown. (DOC) [file pone.0161019.s016.doc]

**Supporting Table 4**: (A) Correlation (r) values are given for the outer and inner stripe width in millimeters (Out-Stri-mm and Inn-Stri-mm) with fibrosis measures, including all of the tissue, the cortex (Ctx), and the medulla (Med) using image analysis of trichrome (Tri). (B) Correlation (r) values are given for the outer and inner stripe width in millimeters (Out-Stri-mm and Inn-Stri-mm) with measures of % epithelial mass by visual assessment. The corresponding P values are also shown.

| **A. Regression r values** | | | | | |
| --- | --- | --- | --- | --- | --- |
|  |  |  |  |  |  |
| **Row** | **Out-Stri-mm** | **Inn-Stri-mm** | **All-Tri** | **Ctx-Tri** | **Med-Tri** |
| Out-Stri-mm | 1.00 | 0.01 | 0.00 | -0.02 | 0.07 |
| Inn-Stri-mm | 0.01 | 1.00 | 0.56 | 0.39 | 0.48 |
| All-Tri | 0.00 | 0.56 | 1.00 | 0.84 | 0.87 |
| Ctx-Tri | -0.02 | 0.39 | 0.84 | 1.00 | 0.68 |
| Med-Tri | 0.07 | 0.48 | 0.87 | 0.68 | 1.00 |
|  |  |  |  |  |  |
| **A. Corresponding p value** | | | | | |
|  |  |  |  |  |  |
| **Row** | **Out-Stri-mm** | **Inn-Stri-mm** | **All-Tri** | **Ctx-Tri** | **Med-Tri** |
| Out-Stri-mm | <0.00001 | 0.94570 | 0.97866 | 0.85386 | 0.61144 |
| Inn-Stri-mm | 0.94570 | <0.00001 | <0.00001 | 0.00170 | 0.00008 |
| All-Tri | 0.97866 | <0.00001 | <0.00001 | <0.00001 | <0.00001 |
| Ctx-Tri | 0.85386 | 0.00170 | <0.00001 | <0.00001 | <0.00001 |
| Med-Tri | 0.61144 | 0.00008 | <0.00001 | <0.00001 | <0.00001 |
|  |  |  |  |  |  |
|  |  |  |  |  |  |
| **B. Regression r values** | | | | | |
|  |  |  |  |  |  |
| **Row** | **Out-Stri-mm** | **Inn-Stri-mm** | **Vis-All-Tri-Epithel** | **Vis-Ctx-Tri-Epithel** | **Vis-Med-Tri-Epithel** |
| Out-Stri-mm | 1.00 | 0.01 | -0.28 | -0.25 | -0.26 |
| Inn-Stri-mm | 0.01 | 1.00 | -0.33 | -0.28 | -0.22 |
| Vis-All-Tri-Epithel | -0.28 | -0.33 | 1.00 | 0.91 | 0.78 |
| Vis-Ctx-Tri-Epithel | -0.25 | -0.28 | 0.91 | 1.00 | 0.67 |
| Vis-Med-Tri-Epithel | -0.26 | -0.22 | 0.78 | 0.67 | 1.00 |
|  |  |  |  |  |  |
| **B. Corresponding p values** | | | | | |
|  |  |  |  |  |  |
| **Row** | **Out-Stri-mm** | **Inn-Stri-mm** | **Vis-All-Tri-Epithel** | **Vis-Ctx-Tri-Epithel** | **Vis-Med-Tri-Epithel** |
| Out-Stri-mm | <0.00001 | 0.93924 | 0.02738 | 0.05066 | 0.03940 |
| Inn-Stri-mm | 0.93924 | <0.00001 | 0.00977 | 0.02641 | 0.09307 |
| Vis-All-Tri-Epithel | 0.02738 | 0.00977 | <0.00001 | <0.00001 | <0.00001 |
| Vis-Ctx-Tri-Epithel | 0.05066 | 0.02641 | <0.00001 | <0.00001 | <0.00001 |
| Vis-Med-Tri-Epithel | 0.03940 | 0.09307 | <0.00001 | <0.00001 | <0.00001 |
