# Supplementary material for: Renal Medullary and Cortical Correlates in Fibrosis, Epithelial Mass, Microvascularity, and Microanatomy Using Whole Slide Image Analysis Morphometry
Source: PLoS One. 2016 Aug 30;11(8):e0161019. doi: 10.1371/journal.pone.0161019 (PMC5004931; doi:10.1371/journal.pone.0161019)
Supplement: S5 Table — The corresponding P values are also shown. (DOC) [file pone.0161019.s017.doc]

Supporting Table 5: Correlation (r) values for measures of microvessel density (MVD in in vessels/um^2^) and mean vessel area (MVA in um^2^) are shown for the all of the tissue, the cortex (Ctx), and the medulla (Med). The corresponding P values are also shown.

| **Regression r values** | |  |  |  |  |  |
| --- | --- | --- | --- | --- | --- | --- |
|  |  |  |  |  |  |  |
|  | **All-MVD** | **All-MVA** | **Ctx-MVD** | **Ctx-MVA** | **Med-MVD** | **Med-MVA** |
| All-MVD | 1.00 | 0.64 | 0.77 | 0.74 | 0.87 | 0.55 |
| All-MVA | 0.64 | 1.00 | 0.63 | 0.83 | 0.55 | 0.87 |
| Ctx-MVD | 0.77 | 0.63 | 1.00 | 0.75 | 0.76 | 0.55 |
| Ctx-MVA | 0.74 | 0.83 | 0.75 | 1.00 | 0.67 | 0.71 |
| Med-MVD | 0.87 | 0.55 | 0.76 | 0.67 | 1.00 | 0.49 |
| Med-MVA | 0.55 | 0.87 | 0.55 | 0.71 | 0.49 | 1.00 |
|  |  |  |  |  |  |  |
| **Corresponding p values** | | |  |  |  |  |
|  |  |  |  |  |  |  |
|  | **All-MVD** | **All-MVA** | **Ctx-MVD** | **Ctx-MVA** | **Med-MVD** | **Med-MVA** |
| All-MVD | <0.00001 | <0.00001 | <0.00001 | <0.00001 | <0.00001 | <0.00001 |
| All-MVA | <0.00001 | <0.00001 | <0.00001 | <0.00001 | <0.00001 | <0.00001 |
| Ctx-MVD | <0.00001 | <0.00001 | <0.00001 | <0.00001 | <0.00001 | <0.00001 |
| Ctx-MVA | <0.00001 | <0.00001 | <0.00001 | <0.00001 | <0.00001 | <0.00001 |
| Med-MVD | <0.00001 | <0.00001 | <0.00001 | <0.00001 | <0.00001 | 0.00003 |
| Med-MVA | <0.00001 | <0.00001 | <0.00001 | <0.00001 | 0.00003 | <0.00001 |
