# Supplementary material for: Renal Medullary and Cortical Correlates in Fibrosis, Epithelial Mass, Microvascularity, and Microanatomy Using Whole Slide Image Analysis Morphometry
Source: PLoS One. 2016 Aug 30;11(8):e0161019. doi: 10.1371/journal.pone.0161019 (PMC5004931; doi:10.1371/journal.pone.0161019)
Supplement: S6 Table — (DOC) [file pone.0161019.s018.doc]

**Supporting Table 6:** Patient demographics, clinical features, and selected biopsy findings are shown.

| Total Number (N) | 67 |
| --- | --- |
| Gender^1^ | 35 (53%) females, 31 (47%) males |
| Age (Mean ± standard deviation) | 58.5 ± 16.9^1^ |
| Age Range^1^ | 6.7 to 89.9 years |
| Creatinine Range^2^ | 0.5 to 23 mg/dL |
| Creatinine (Mean ± standard deviation) ^2^ | 2.1 ± 3.4 mg/dL |
| Biopsy Indication | Increased creatinine (N = 28), Increased creatinine and proteinuria (N = 9), Proteinuria (N = 7), Nephrotic syndrome (N = 4), Hematuria and Proteinuria (N = 3), Nephrectomy for carcinoma (N = 3), Increased creatinine and nephrotic range proteinuria (N = 2), Proteinuria (N = 2), Hematuria (N = 2), Increased creatinine and hematuria (N = 1), Nephrotic range proteinuria (N = 1), Nephrotic syndrome (N = 1), and unclear or unavailable indication (N = 4) |

| Glomerulosclerosis, global | Mean ± standard deviation = 29 ± 23%, Range = 0 to 80% |
| --- | --- |
| Glomerulosclerosis, segmental | Mean ± standard deviation = 1.8 ± 6.8%, Range = 0 to 43% |
| Diagnosis | 17 glomerulosclerosis of uncertain etiology  14 diabetic glomerulosclerosis  6 IgA nephropathy  4 Focal Segmental Glomerulosclerosis  5 Lupus nephritis  4 Tubulointerstitial nephritis  3 Acute tubular injury/necrosis  3 Non-neoplastic parenchyma from nephrectomy for carcinoma  3 No diagnostic abnormality recognized or nonspecific findings  2 Immune complex glomerulonephritis (GN)  1 each of the following: amyloidosis, granulomatous interstitial nephritis, light chain deposition disease, minimal change disease, membranous GN, membranoproliferative GN due to monoclonal immunoglobulin deposition disease |

^1^ Data was not available in 1 patient.

^2^Concurrent creatinine data was available in 50 patients.
