# Supplementary material for: Renal Medullary and Cortical Correlates in Fibrosis, Epithelial Mass, Microvascularity, and Microanatomy Using Whole Slide Image Analysis Morphometry
Source: PLoS One. 2016 Aug 30;11(8):e0161019. doi: 10.1371/journal.pone.0161019 (PMC5004931; doi:10.1371/journal.pone.0161019)
Supplement: S7 Table — (DOC) [file pone.0161019.s019.doc]

**Supporting Table 7**: Correlation r values are shown in order of strength of association for the measurements that had “very high” and “high” correlations between cortex and medulla^.

| **Variable** | **by Variable** | **Correlation r value*** |
| --- | --- | --- |
|  |  |  |
| **“Very high” correlations** | | |
| Med-RedTri | Med-Tri | -0.95 |
| Ctx-RedTri | Ctx-Tri | -0.94 |
|  |  |  |
| **“High” correlations** | | |
| Med-T-P | Med-Tri | 0.89 |
| Med-PAS | Ctx-PAS | 0.87 |
| Vis-Med-PAS-TA | Vis-Ctx-PAS-TA | 0.86 |
| Med-CKAvg | Ctx-CKAvg | 0.85 |
| Ctx-T-P | Ctx-Tri | 0.84 |
| Med-T-P | Med-RedTri | -0.83 |
| Ctx-Col | Med-PAS | -0.81 |
| Ctx-Col | Ctx-PAS | -0.81 |
| Med-MVD | Ctx-MVD | 0.76 |
| Ctx-MVA | Ctx-MVD | 0.75 |
| Ctx-T-P | Ctx-RedTri | -0.75 |
| Med-T-P | Ctx-T-P | 0.72 |
| Vis-Ctx-Tri-Epithel | Vis-Ctx-PAS-TA | -0.71 |
| Med-MVA | Ctx-MVA | 0.71 |
| Ctx-Col | Ctx-MVD | 0.70 |
| Vis-Ctx-Tri-Epithel | Vis-Ctx-Tri | -0.70 |

^ Interstitial fibrosis, tubular atrophy, epithelial cell mass [EPCM, Epithel below], microvessel density (MVD), and microvessel area (MVA) were measured. Stains applied include trichrome (Tri), periodic acid–Schiff (PAS), collagen III (Col) immunohistochemistry (IHC), cytokeratin (CK) IHC for EPCM, and CD34 IHC for the MVD and MVA. Measurements are either performed using morphometric or visual (Vis) methods. For fibrosis morphometry, a method employing trichrome minus the PAS measurement is used (T-P). For EPCM, the “Red” of the trichrome is also used (RedTri).

* For all correlation r values, P ≤ 0.0002.
